# Thermal Treatment Effect on CO and NO Adsorption on Fe(II) and Fe(III) Species in Fe_3_O-Based MIL-Type Metal–Organic Frameworks: A Density Functional Theory Study

**DOI:** 10.1021/acs.inorgchem.1c01044

**Published:** 2021-06-10

**Authors:** Jenny G. Vitillo, Laura Gagliardi

**Affiliations:** † Department of Science and High Technology and INSTM, University of Insubria, Via Valleggio 9, 22100 Como, Italy; ∥ Department of Chemistry, Chemical Theory Center, and Supercomputing Institute, University of Minnesota, 207 Pleasant Street S.E., Minneapolis, Minnesota 55455-0431, United States; ‡ Department of Chemistry, Pritzker School of Molecular Engineering, James Franck Institute, University of Chicago, Chicago, Illinois 60637, United States

## Abstract

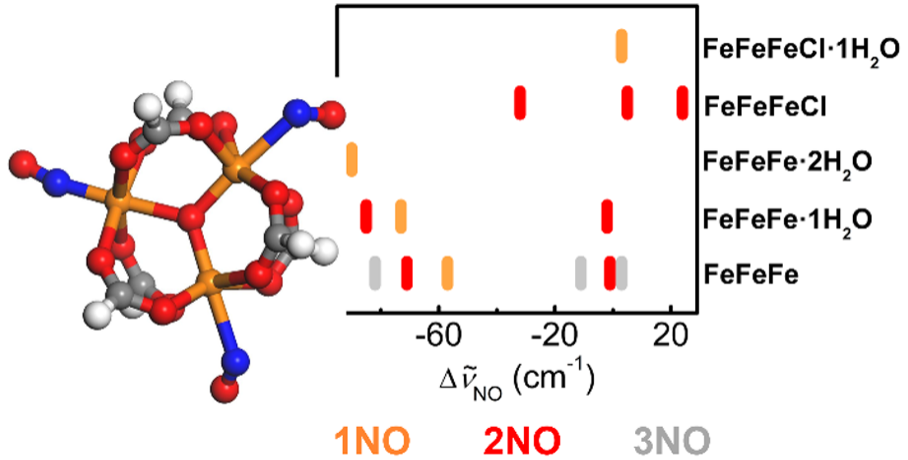

The properties of metal–organic frameworks (MOFs) based on triiron oxo-centered (Fe_3_O) metal nodes are often related to the efficiency of the removal of the solvent molecules and the counteranion chemisorbed on the Fe_3_O unit by postsynthetic thermal treatment. Temperature, time, and the reaction environment play a significant role in modifying key features of the materials, that is, the number of open metal sites and the reduction of Fe(III) centers to Fe(II). IR spectroscopy allows the inspection of these postsynthetic modifications by using carbon monoxide (CO) and nitric oxide (NO) as probe molecules. However, the reference data sets are based on spectra recorded for iron zeolites and oxides, whose structures are different from the Fe_3_O one. We used density functional theory to study how the adsorption enthalpy and the vibrational bands of CO and NO are modified upon dehydration and reduction of Fe_3_O metal nodes. We obtained a set of theoretical spectra that can model the modification observed in previously reported experimental spectra. Several CO and NO bands were previously assigned to heterogeneous Fe(II) and Fe(III) sites, suggesting a large defectivity of the materials. On the basis of the calculations, we propose an alternative assignment of these bands by considering only crystallographic iron sites. These findings affect the common description of Fe_3_O-based MOFs as highly defective materials. We expect these results to be of interest to the large community of scientists working on Fe(II)- and Fe(III)-based MOFs and related materials.

## Abstract 2

Thermal treatment of triiron oxo-centered (Fe_3_O)-based metal−organic frameworks is a common postsynthetic method to determine the material performances in many applications: we used density functional theory methods to study how the efficacy of the treatment modifies the energetics and the vibrational bands of nitric oxide (NO) and carbon monoxide. The obtained data set is meant to be part of the characterization toolboxes aimed at the assessment of thermal treatment protocols.

## 1 Introduction

**Figure 1:**
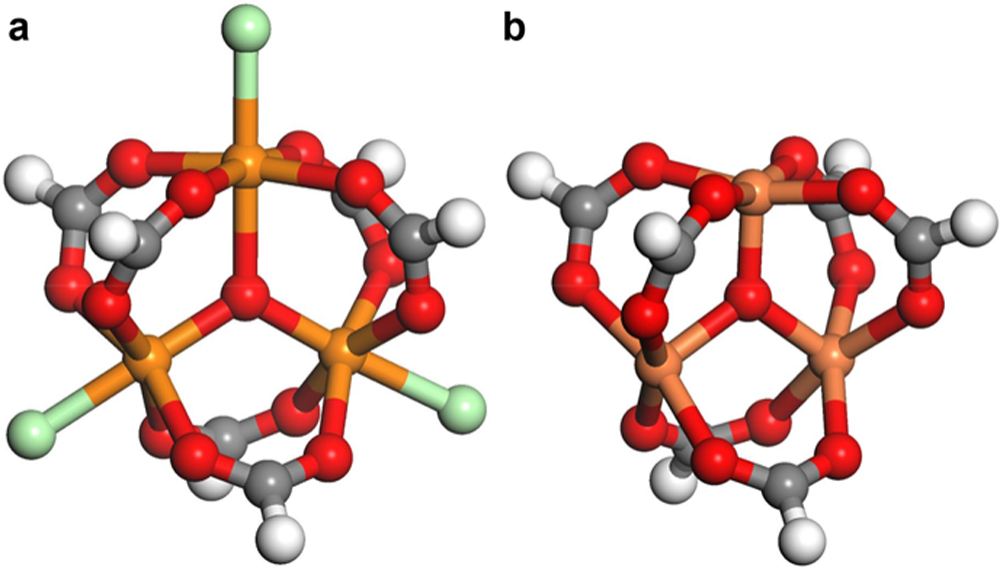
Models of the triiron oxo-centered metal clusters formed upon thermal treatment in MIL-100(Fe) at different temperatures (all lower than the decomposition temperature of the MOF): (a) [Fe^III^_3_(μ_3_-O)(X)(H_2_O)_2_](HCOO)_6_ (Fe_3_O-X·2H_2_O) as a model of the as-synthesized material; (b) [Fe^II^Fe^III^_2_(μ_3_-O)](HCOO)_6_ (Fe_3_O) model of the fully activated material, where the counterion (X) and all of the water molecules have been removed. Structures are optimized at the UM06-L/def2-TZVP level. All of the other clusters can be obtained from part a by the removal of X and/or water. Color code: green, water or X; red, oxygen; gray, carbon; orange, iron; white, hydrogen.

Metal–organic frameworks (MOFs) based on a triiron oxo-centered cluster (Fe_3_O)^1,2^ are important materials for several applications, including gas storage and separation,^3−5^ heat pump applications,^6,7^ catalysis,^8−10^ and drug delivery.^1,11,12^ The most representative of this class of MOFs are MIL-100(Fe)^2^ and MIL-101(Fe) (MIL = Materials Institute Lavoisier).^13^ In the as-synthesized material, the metal node has the formula [Fe^III^_3_(μ_3_-O)(X)(Z)_2_]^6+^ (Figure 1a), where Z is a solvent molecule (e.g., water) and X is a counteranion.^5^ The anion originates from the reagents used in the MOF synthesis (e.g., −OH from NaOH or KOH, −F from HF, and −Cl from iron chloride salts). More than one kind of X and Z can be present in the same material.

Upon heating of the material in a vacuum or in a flow of inert gas, both the X and Z species can be removed from the metal node, creating an open iron site that can coordinate an adsorbate.^5^ The oxidation state of the iron centers does not change upon the removal of Z. The removal of X, instead, causes the reduction of one Fe(III) to Fe(II). This means that upon increasing the temperature we can go from the as-synthesized sample, having all of the nodes with the formula [Fe^III^_3_(μ_3_-O)(X)(Z)_2_]^6+^ (Figure 1a), to a fully activated MOF with [Fe^II^Fe^III^_2_(μ_3_-O)]^6+^ metal nodes (Figure 1b). At intermediate temperatures, clusters with different compositions coexist, having the general formula [Fe^II^_*x*_Fe^III^_*y*_(μ_3_-O)(X)_*l*_(Z)_*n*_]^6+^, with *x* = 0 and 1, *y* = 3 – *x*, *l* = 1 – *x*; 0 ≤ *n* ≤ 2 + *x*. The presence of open iron centers and, in particular Fe(II) sites, has been considered to be one of the reasons for the successful performance of Fe_3_O-based MOFs in catalysis^8,9^ and gas separation.^3−5^ It is then important to evaluate the efficacy of the possible postsynthetic protocols in order to tailor the number of open Fe(II) and Fe(III) species for the different purposes. Vibrational and Mössbauer spectroscopies^4,5,9,14^ and microcalorimetry^4^ are commonly used to optimize postsynthetic protocols involving the iron centers as the thermal treatment of these materials and the grafting of guest species.^15^ IR spectroscopy using nitric oxide (NO) and carbon monoxide (CO) as molecular probes is particularly suitable for the characterization of iron-based materials because the stretching frequency of these molecules is shifted to different spectral regions if they are coordinated to Fe(II) or Fe(III) centers.^16,17^ IR spectroscopy of CO and NO is an efficient diagnostic tool to determine the iron oxidation state in MOF materials. Previous spectroscopic studies on MIL-100(Fe) suggested the presence of heterogeneous Fe(II) and Fe(III) sites.^5,14^ Nevertheless, no explanation of the origin of this heterogeneity was provided besides its possible correlation with the presence of an unreacted linker^18^ in the material and the presence of defects.^5^ In fact, the only data that can be used for the assignment of the NO and CO bands in IR spectra in Fe-based MOFs are based on studies on oxides and zeolites,^5,14^ whose structures are different compared to that of the Fe_3_O cluster. The study of CO and NO adsorption on Fe_3_O-based MOFs is relevant to gas separation and therapeutics. NO removal from exhaust gases is an important process.^19^ CO removal from H_2_ and CH_4_ is a mandatory step for their safe use in fuel cells.^4,20^ NO^21−23^ and CO^22^ are also important gasotransmitters in physiological and biological functions. MIL-100(Fe) and MIL-101(Fe) are among the most studied MOFs for drug delivery because of their high capacity and low toxicity.^1,11,12^ Understanding how the presence of water modifies the interaction of NO and CO with the drug carrier is pivotal for these applications because water triggers the gas release.^4^ Moreover, the effect of water on NO and CO adsorption in MOFs is important because water is often present as a contaminant in many gas mixtures and can affect the separations.

In this study, we used Kohn–Sham density functional theory (DFT) methods to determine if the presence of water molecules and of X influences the adsorption enthalpy and stretching frequency of CO and NO in Fe_3_O-based materials. The effect of CO and NO coverage was also evaluated. We considered two X, −OH and −Cl, to assess the vibrational shift of the molecular probes. We adopted cluster models, namely, [Fe^II^_*x*_Fe^III^_*y*_(μ_3_-O)(X)_*l*_(Z)_*n*_]^6+^ units coordinated to six formates. Seven different models were considered: [Fe^II^Fe^III^_2_(μ_3_-O)](HCOO)_6_ (Fe_3_O in the following; see Figure 1b), [Fe^II^Fe^III^_2_(μ_3_-O)(H_2_O)](HCOO)_6_ (Fe_3_O·1H_2_O), [Fe^II^Fe^III^_2_(μ_3_-O)(H_2_O)_2_](HCOO)_6_ (Fe_3_O·2H_2_O), [Fe^III^_3_(μ_3_-O)Cl](HCOO)_6_ (Fe_3_O-Cl), [Fe^III^_3_(μ_3_-O)(OH)](HCOO)_6_ (Fe_3_O-OH), [Fe^III^_3_(μ_3_-O)Cl(H_2_O)](HCOO)_6_ (Fe_3_O-Cl·1H_2_O), and [Fe^III^_3_(μ_3_-O)(OH)(H_2_O)](HCOO)_6_ (Fe_3_O-OH·1H_2_O). On the basis of our results, we propose an alternative assignment to the one generally accepted in the literature of the IR bands of CO^4,5,14^ and NO^14,24^ adsorbed in Fe_3_O-based MOFs by considering only crystallographic sites.

## 2 Computational Methods

DFT calculations were performed using the M06-L functional^25^ in its unrestricted formalism (U) in combination with the def2-TZVP basis sets,^26,27^ as implemented in the *Gaussian 16* program.^28^ Previous investigations showed that this level of theory correctly reproduces the electronic properties of iron centers in MOFs,^29^ in particular Fe_3_O, when benchmarked versus multireference calculations.^8^ The Fe_3_O model has been previously employed to describe N_2_O reactivity on MIL-100.^9^ Moreover, this model has been used by Mavrandonakis et al.^30^ to predict the adsorption enthalpies and vibrational frequencies of different adsorbates in trimetal oxo-centered MOFs. This model has shown results similar to those reported for a cluster coordinated to benzoate instead of formate groups in reactivity^31^ and in adsorption studies.^30^

Geometry optimizations were carried out by means of the Berny optimization algorithm with an analytical gradient. A (99, 590) pruned grid was used (i.e., 99 radial points and 590 angular points per radial point). The *Gaussian 16* default convergence thresholds were set for optimization. All of the energetic data were corrected for basis set superposition error (BSSE) following the a posteriori method proposed by Boys and Bernardi,^32^ as implemented in *Gaussian 16*. The energy and enthalpy of adsorption for the complex with (*n*+1) L molecules are defined as
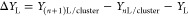
The BSSE-corrected values, indicated by a c superscript, were obtained from the computed *Y* values as
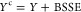


Unscaled harmonic frequencies were obtained analytically. Enthalpies and Gibbs free energies were calculated at 1 atm and 298 K using the scheme proposed by De Moor et al.,^33^ whereby low-lying frequency modes (<50 cm^–1^) were replaced by a cutoff value (50 cm^–1^) in the calculation of the vibrational partition functions.^34−38^ Charge and spin densities were obtained using Charge Model 5 (CM5)^39^ and Hirshfeld population analysis,^40^ respectively. Spin densities are expressed as the difference between the α and β electron densities.

## 3 Results and Discussion

The dependence on the temperature of the water and X [or Fe(II)] content in Fe_3_O-based MOFs was determined experimentally in previous studies.^4,5^ We summarize prior results to guide the reader in a comparison between our computational results and the experimental ones. Leclerc et al.^5^ treated MIL-100(Fe) (X = 81% F, OH, trimesate) in a dynamic vacuum in the 25–300 °C range: they observed the complete removal of free and bonded water at 150 °C, with the formation of only a small fraction of Fe(II). Above 150 °C, the concentration of Fe(II) increases with the temperature: Yoon et al.^4^ reported a maximal removal of 40% of the initial Fe–X sites at 260 °C. Above 260 °C, the sample starts to decompose. A slightly different behavior was reported by Wuttke et al.^14^ using a helium flow: at 150 °C, they observed only the removal of physisorbed water, while the water directly bonded to the Fe_3_O clusters was fully desorbed at 200 °C. Moreover, the removal of X is far less effective in a helium flow than in dynamic vacuum.^4^ Also X plays a role in the thermal behavior of Fe_3_O samples: the removal of only 4–5% of Fe–X was reported at 250 °C for a MIL-100(Fe) (X = 20% Cl, OH, trimesate) sample.^9,18^ Different clusters have to be adopted to model MIL-100(Fe) treated at different temperatures *T* in degrees Celsius [in the following MIL-100(Fe)-*T*C] because the type and number of adsorbed species (X and Z) on the metal node are different. Considering the information reported in refs (4) and (5), we have used Fe_3_O-Cl·2H_2_O, Fe_3_O-OH·2H_2_O, and Fe_3_O·3H_2_O to model MIL-100(Fe) samples treated at *T* ≤ 100 °C. Fe_3_O-Cl·1H_2_O, Fe_3_O·1H_2_O, Fe_3_O-OH·1H_2_O, and Fe_3_O·2H_2_O have been used for MIL-100(Fe)-120C. For MIL-100(Fe) treated at 150, 200, and 250 °C, the models used have been the same (Fe_3_O-Cl, Fe_3_O-OH, and Fe_3_O), with only the proportion of the species being different (e.g., Fe_3_O represents 2% of all of the metal nodes in MIL-100(Fe)-150C and 40% of the metal nodes in MIL-100(Fe)-250C treated in a dynamic vacuum for 40 h).

The ground-state electronic configuration for all of the systems has the three iron centers in high spin states. However, the most stable configuration for the Fe_3_O-Cl, Fe_3_O-OH, and Fe_3_O is not the highest possible spin state for the cluster (HS) but the “broken-symmetry” solution (BS) where two high-spin Fe(III) centers couple antiferromagnetically. Although the BS energetics would be more accurate,^9,41^ the corresponding wave function is not a spin eigenfunction nor does it have the correct spin density. Moreover, the BS solution is strongly dependent on the initial guess, hindering both the reproducibility of the BS results and the comparison among different studies. Following a common strategy,^41−43^ we modeled all of the clusters considering the Fe_3_O node in HS. In general, the difference in energy between the HS and BS is 20–30 kJ mol^–1^. For details on this choice, see the discussion reported in previous studies.^8,9,31^ Coordination of the adsorbates can cause a change in the ground spin state of the iron centers. This has been evaluated for a range of spin states starting from the HS value of the bare triiron oxo-centered clusters to determine the most stable spin state.

The calculations indicate that each iron center can coordinate only one adsorbate molecule: when more than one molecule is adsorbed on one metal node, each molecule is coordinated to a different metal site of the metal node. The highest coverage corresponds to filling of the position left free by X and Z (marked with green spheres in Figure 1a). This agrees with the available IR experiments on CO and NO adsorption that show the formation of monocarbonyls and mononitrosyls only.^5,14^ Only in the NO case, forcing the formation of a Fe···2NO complex brings displacement of the carboxylate groups. This complex is a local minimum, but it is less stable than the mononitrosyl complex by 10 kJ mol^–1^. Such a displacement is possible in a cluster model where no geometrical constraints were used in the optimization, while it is unlikely to happen in the MOF structure because of the framework constraints and because of the large cluster distortion, at least at subatmospheric NO pressures considered in the experiments.^24^ Accordingly, the experimental spectra do not show the formation of dinitrosyls.^24^

### H_2_O Adsorption

**Figure 2:**
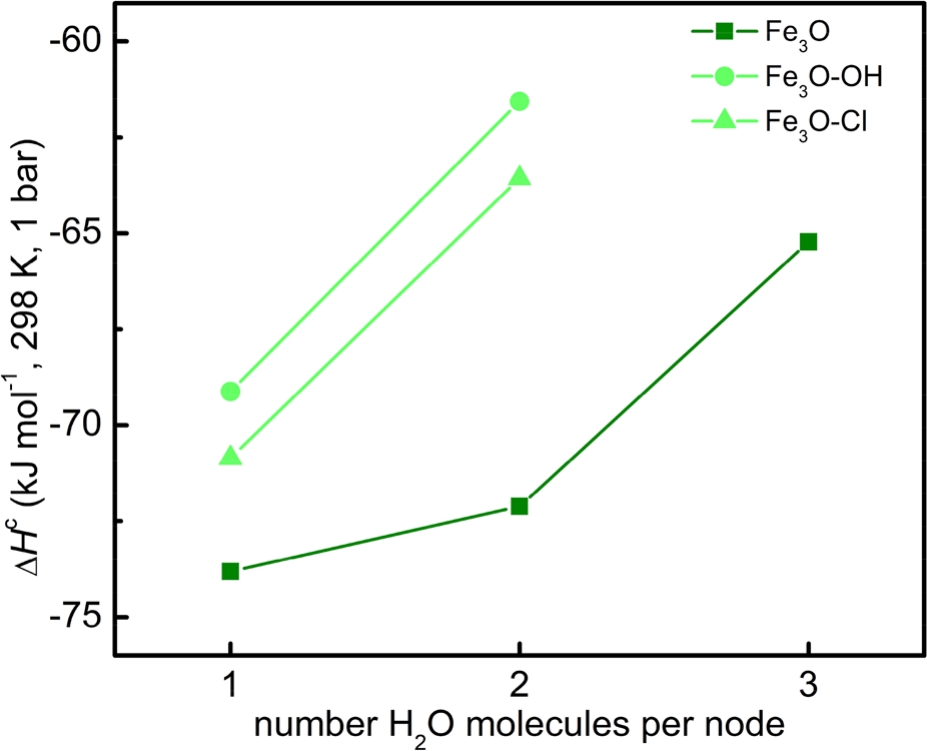
Enthalpy of water adsorption on Fe_3_O clusters, Δ*H*^c^, as a function of the water coverage, obtained at the UM06-L/def2-TZVP level for the three models of the fully dehydrated MOFs (treatment temperature ≥150 °C): Fe_3_O (green squares), Fe_3_O-Cl (light-green triangles), and Fe_3_O-OH (light-green circles).

The water adsorption on Fe_3_O, Fe_3_O-OH, and Fe_3_O-Cl clusters was studied by increasing the number of water molecules until all of the open iron sites are coordinated. The results are reported in Table 1, Figure 2, and Table S6.

**Table 1:** Water Adsorption on Fe_3_O Clusters Optimized at the UM06-L/def2-TZVP Level in Their Ground Spin State (*S*)^a^

model	2*S* + 1	*d*(Fe–O_H_2_O_)	*d*(Fe–O_c_)	Δ*E*_H_2_O_	Δ*E*_H_2_O_^c^	Δ*H*_H_2_O_^c^	Δ*G*_H_2_O_^c^
Fe_3_O							
1H_2_O	15	2.169	1.921	–86.9	–81.8	–73.8	–29.2
2H_2_O	15	2.185	1.919	–85.5	–79.5	–72.1	–30.6
		2.184	1.919				
3H_2_O	15	2.201	1.913	–79.5	–73.5	–65.2	–19.5
		2.200	1.907				
		2.204	1.906				
Fe_3_O-Cl							
1H_2_O	16	2.196	1.891	–83.4	–77.0	–69.6	–27.3
2H_2_O	16	2.217	1.879	–77.4	–71.2	–63.6	–20.6
		2.221	1.883				
Fe_3_O-OH							
1H_2_O	16	2.198	1.888	–82.9	–76.6	–69.1	–25.4
2H_2_O	16	2.223	1.876	–76.0	–69.8	–61.6	–17.1
		2.223	1.880				

a All of the reported values refer only to the iron sites coordinating a water molecule. The distance of the reacting iron from the water O *d*(Fe–O_H_2_O_) and from the central O of the Fe_3_O cluster *d*(Fe–O_c_) are also reported (in angstroms). The BSSE-corrected adsorption energy Δ*E*_H_2_O_^c^, adsorption enthalpy Δ*H*_H_2_O_^c^, and adsorption Gibbs free energy Δ*G*_H_2_O_^c^ are reported in kilojoules per mole. The value not corrected for the BSSE is also shown for the energy (Δ*E*_H_2_O_). *H* has been calculated at 1013.25 mbar and 25 °C.

All of the values reported in Table 1 for the adsorption enthalpy Δ*H*_H_2_O_^c^ are within the range from −74 to −62 kJ mol^–1^. The calculations show that Δ*H*_H_2_O_^c^ has only a slight dependence on the iron oxidation state [it increases upon going from Fe(II) to Fe(III)], while its dependence on the counteranion is negligible (it increases upon going from −Cl to −OH; Table 1 and Figure 2). The Δ*H*_H_2_O_^c^ value becomes less negative with coverage only where the adsorption of the last water molecule is concerned, by about 10 kJ mol^–1^ less than the first one(s).

Jeremias et al.^6^ determine the isosteric heat for water in MIL-100(Fe), after degassing the sample at 120 °C for 17 h. On the basis of refs (5) and (14), this sample should be mainly constituted of Fe_3_O-X clusters, with some residual Fe_3_O-X·1H_2_O and a negligible amount of Fe_3_O. The lowest coverage values reported in ref (6) correspond to about two water molecules per Fe_3_O, and they are characterized by a heat of 90 ± 30 and 80 ± 20 kJ mol^–1^. The isosteric heat then reaches a constant value of 60 ± 10 kJ mol^–1^ for coverage ≥4 H_2_O/Fe_3_O. All of these experimental values are similar, considering the error bar. The values reported in Table 1 and Figure 1 agree with the experimental values and reproduce the small dependence on the coverage of the isosteric heat on MIL-100(Fe).

The vibrational bending modes of water, δ(H_2_O), can be used to determine the oxidation state of the metal node: the calculations indicate that δ(H_2_O) is shifted to lower wavenumbers if the cluster is in its oxidized form, while it is shifted to higher wavenumbers if it is reduced (Figure S3).

### CO Adsorption

Relevant energetic, geometric, and spectroscopy parameters for all of the CO complexes are reported in Table 2, while additional parameters are listed in Tables S2 and S5.

**Table 2:** CO Adsorption on Fe_3_O Clusters^a^

model	2*S* + 1	*d* (Fe–C_CO_)	∠Fe–C–O	Δ*E*_CO_	Δ*E*_CO_^c^	Δ*H*_CO_^c^	Δ*G*_CO_^c^	Δ*ṽ*_CO_
Fe_3_O								
1CO	15	2.316	178	–46.5	–49.6	–45.3	–7.8	29
2CO	15	2.385	178	–43.4	–46.4	–41.9	–3.5	45
		2.385	178					45
3CO	15	2.412	179	–41.8	–44.8	–40.2	–1.7	48
		2.408	179					47
		2.412	180					46^b^
Fe_3_O·1H_2_O								
1CO	15	2.383	178	–44.6	–47.6	–43.3	–5.6	45
2CO	15	2.416	179	–40.7	–37.8	–33.2	4.9	44
		2.416	179					43
Fe_3_O·2H_2_O								
1CO	15	2.426	180	–39.9	–37.0	–32.7	5.0	44
Fe_3_O-Cl								
1CO	16	2.428	180	–42.8	–39.6	–35.3	2.5	51
2CO	16	2.448	180	–39.0	–35.9	–31.5	5.8	46
		2.451	179					45
Fe_3_O-Cl·1H_2_O								
1CO	16	2.462	180	–38.7	–35.6	–31.4	5.7	44
Fe_3_O-OH								
1CO	16	2.425	180	–42.1	–38.9	–34.6	3.8	50
2CO	16	2.445	179	–38.4	–35.3	–30.8	6.9	45
		2.445	179					44
Fe_3_O-OH·1H_2_O								
1CO	16	2.456	179	–37.7	–34.6	–30.3	7.9	43

a All of the values refer only to the iron sites coordinating a CO molecule. Clusters were optimized at the UM06-L/def2-TZVP level in their ground spin state (*S*). The distance of the reacting iron from the C of the CO molecule [*d*(Fe–C_CO_) in angstroms] and the Fe···CO angle (∠Fe–C–O, in degrees) are also reported. The stretching frequency shift (Δ*ṽ*_CO_ in reciprocal centimeters) is calculated from the gas-phase values (*ṽ*_CO_ = 2202 cm^–1^). The BSSE-corrected adsorption energy Δ*E*_CO_^c^, adsorption enthalpy Δ*H*_CO_^c^, and adsorption Gibbs free energy Δ*G*_CO_^c^ are reported in kilojoules per mole. The value not corrected for the BSSE is also shown for the energy (Δ*E*_CO_). *H* and *G* have been calculated at 1013.25 mbar and 25 °C.

b Vibration involving only one CO molecule (made explicit only for the 2CO and 3CO complexes).

**Figure 3:**
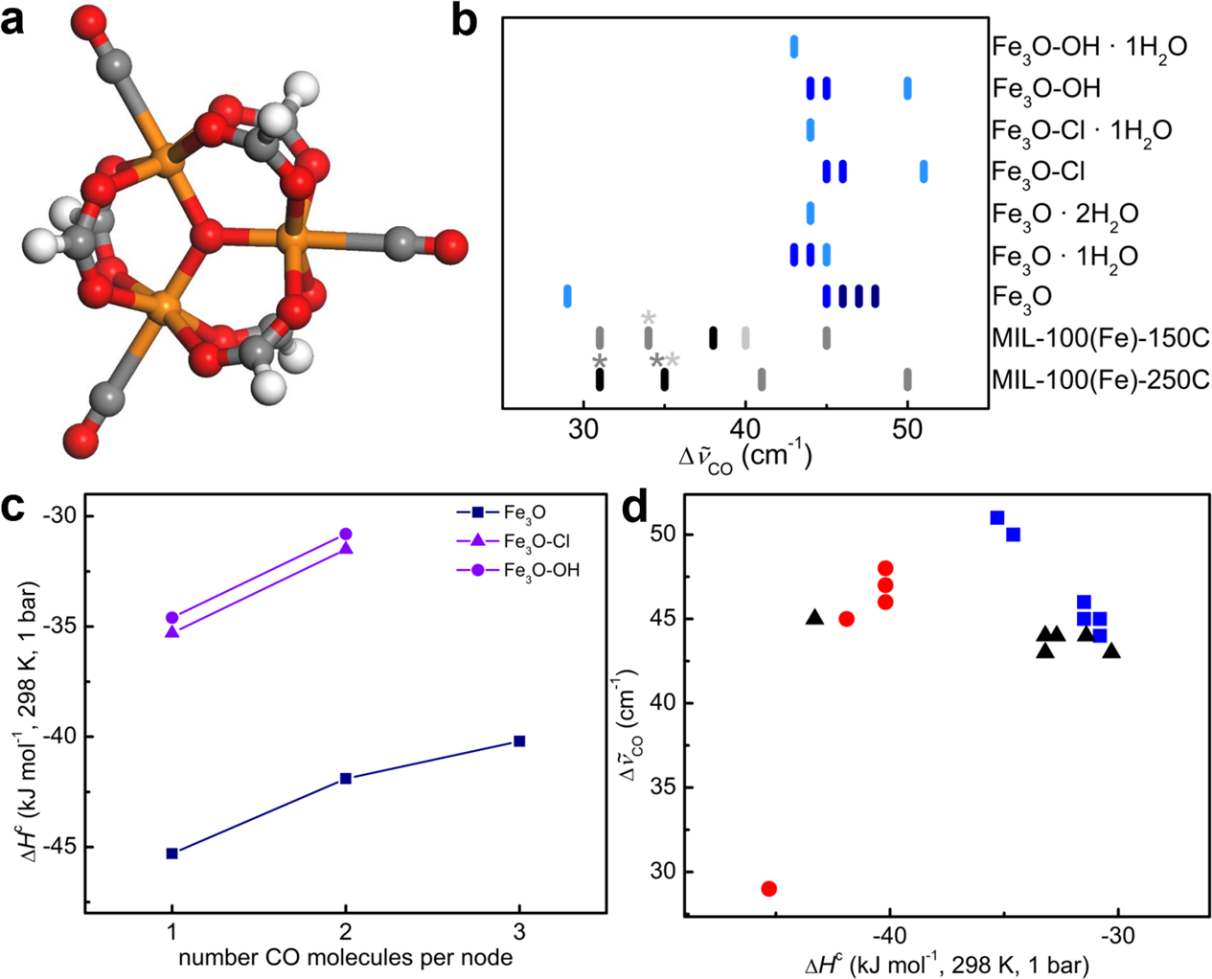
CO complexes on Fe_3_O clusters. (a) Optimized structure of 3CO/Fe_3_O. The color code is as in Figure 1. Results were obtained at the UM06-L/def2-TZVP level and are reported in Table 2. (b) CO vibrational shifts on Fe_3_O, Fe_3_O-OH, and Fe_3_O-Cl for different degrees of hydration of the metal node. The colors differentiate different loadings of CO per metal node, and then the CO species that can be formed at different pressures. Color code: light blue, 1CO complexes; blue, 2CO; dark blue, 3CO. In all of the complexes, the minimum geometries show the formation of only monocarbonyl species. The formation of dicarbonyl or tricarbonyl is not allowed. The data in ref (5) for ∼zero (light-gray lines), low-to-middle (gray), and high (black) pressures, as in Table 3, for MIL-100(Fe)-150C and MIL-100(Fe)-250C are also reported. The asterisks indicate the bands that are also associated with the ∼0 (light gray) or low-to-middle (gray) coverage. (c) Enthalpy of adsorption on Fe_3_O clusters, Δ*H*^c^, as a function of the coverage on models for the fully dehydrated MOFs (treatment temperature ≥150 °C): Fe_3_O, blue squares; Fe_3_O-Cl, violet triangles; Fe_3_O-OH, violet circles. (d) Dependence of the CO stretching frequency shift Δ*ṽ*_CO_ on the adsorption enthalpy Δ*H*_CO_^c^ for the clusters modeling Fe_3_O-based materials treated at temperature lower (black triangles) or higher (blue squares, Fe_3_O-Cl and Fe_3_O-OH; red circles, Fe_3_O) than 150 °C. Complexes with lower adsorption enthalpies correspond to species formed at lower equilibrium pressures and at higher temperatures in the experiments (Table 3).

CO is adsorbed end-on, C-side, in all of the complexes on the iron centers, with a linear geometry (Table 2 and Figure 3a). The enthalpy of adsorption, Δ*H*_CO_^c^, spans a range from −45.3 to −30.3 kJ mol^–1^, and it is lower for the Fe_3_O clusters than for the Fe_3_O-OH and Fe_3_O-Cl ones. Moreover, Δ*H*^c^ is almost independent on the coverage except for Fe_3_O·1H_2_O, where an increase of 10 kJ mol^–1^ is observed upon going from 1CO/Fe_3_O·1H_2_O to 2CO/Fe_3_O·1H_2_O. The Δ*H*_CO_^c^ values are all lower than the calculated adsorption enthalpies for water (Table 1). This is a valuable property for drug delivery because CO release can be triggered by water. Nevertheless, this is detrimental for CO capture because water would be preferably adsorbed over CO on Fe_3_O-based MOFs. Δ*ṽ*_CO_ is almost constant for all of the complexes in Table 2, with all of the values being between 43 and 51 cm^–1^, with one outlier: the complex of one CO molecule with Fe_3_O (Δ*ṽ*_CO_ = 29 cm^–1^).

Yoon et al.^4^ employed microcalorimetry to study CO adsorption on MIL-100(Fe)-100C and MIL-100(Fe)-250C at 30 °C for pressures from 0 to 150 mbar. Calorimetric values for CO adsorption on MIL-100(Fe)-150C are reported in ref (5). The analysis of these data shows that the heat of adsorption increases with the temperature used for the postsynthetic treatment. For MIL-100(Fe)-100C, the heat of adsorption was found to vary from −34.7 to −29.1 kJ mol^–1^ for coverage as low as 0.013–0.027 CO per Fe_3_O. The heat of adsorption increases to 38–40 kJ mol^–1^ for MIL-100(Fe)-150C,^5^ while for MIL-100(Fe)-250C, it goes from −50.2 kJ mol^–1^ (at 0.047 CO per Fe_3_O) to −39.5 kJ mol^–1^ (at 0.26 CO per Fe_3_O). This dependence was explained by the removal of an increasing number of X and Z from the clusters, and the present calculations support this hypothesis. In MIL-100(Fe)-100C, both X and Z are still coordinated to the Fe_3_O nodes (see section 3). Accordingly, these values are closer to the calculated Δ*H*_CO_^c^ for all of the Fe_3_O-X·*n*H_2_O clusters and for Fe_3_O·2H_2_O (Table 2). In MIL-100(Fe)-150C, Z is fully removed: CO can interact with open Fe^3+^ sites.^5^ The Δ*H*_CO_^c^ values for CO complexes with Fe_3_O-X clusters are close to the experimental values. In MIL-100(Fe)-250C, Z and X are desorbed from the metal nodes, allowing the formation of divalent iron centers.^4,5^ This explains the higher heat of adsorption measured for this sample: Fe(II) sites stabilize the adsorbed CO by π interaction, in addition to the σ-donor interaction with the Fe^3+^ sites.^5^ The calculated Δ*H*_CO_^c^ values for the adsorption of the first CO molecule on Fe_3_O and Fe_3_O·1H_2_O are the closest values to the heat measured for CO adsorption on MIL-100(Fe)-250C.

CO adsorption on MIL-100(Fe) has been investigated by means of IR spectroscopy in previous works.^4,5,14^ The experimental studies have discussed only the changes observed in the CO stretching frequency region. Additional bands associated with vibrational modes involving CO (e.g., the bending mode of Fe···CO) are expected in the spectral region below 800 cm^–1^. The description of theoretical spectra in this range is reported in Figure S1. Yoon et al.^4^ and Wuttke et al.^14^ have studied it at room temperature in a flow of 10% CO in helium (Table 3) to determine how the CO surface species formed at a certain CO partial pressure change with the treatment temperature. Leclerc et al.^5^ have investigated how the CO bands change with the coverage up to CO condensation: this allowed them to characterize all of the adsorption sites present on the MIL surface. Leclerc et al.^5^ have recorded the spectra on MIL-100(Fe)-150C and MIL-100(Fe)-250C at −173 °C in static conditions and by increasing the pressure up to 0.53 mbar (Table 3).

**Table 3:** Review of Experimental CO Stretching Frequencies (*ṽ*_CO_) Recorded for CO Adsorption in MIL-100(Fe) Samples by IR spectroscopy^a^

material	treatment	*T*_*IR*_	*P*_IR_	*ṽ*_CO_^b^	Δ*ṽ*_CO_	original assignment
MIL-100(Fe)^4^	100 °C, 12 h	25	100^c^	2190(l)	47	Fe(III)···CO
	150 °C, 12 h	25	100^c^	2190(l)	47	Fe(III)···CO
				2182(s)	39	Fe(II)···CO
				2173(s)	30	Fe(II)···CO
	200 °C, 12 h	25	100^c^	2190(s)	47	Fe(III)···CO
				2182	39	Fe(II)···CO
				2173(s)	30	Fe(II)···CO
	250 °C, 12 h	25	100^c^	2182(s)	39	Fe(II)···CO
				2173	30	Fe(II)···CO
MIL-100(Fe)^14^	100 °C, 12 h	25	100^c^	2189(l)	46	Fe(III)···CO
	150 °C, 12 h	25	100^c^	2189(l)	46	Fe(III)···CO
				2182(s)	39	Fe(II)···CO
	200 °C, 12 h	25	100^c^	2189(s)	46	Fe(III)···CO
				2182	39	Fe(II)···CO
				2173(s)	30	Fe(II)···CO
	250 °C, 12 h	25	100^c^	2182	39	Fe(II)···CO
				2175	32	Fe(II)···CO
MIL-100(Fe)^5^	150 °C, 12 h	–173	∼0.0	2175(s)	40	Fe(II)···CO
				2169(l)	34	Fe(II)···CO
			low^d^	2192(l)	57	Fe(III)···CO
				2169	34	Fe(II)···CO
			medium^d^	2180(l)	45	Fe(III)···CO
				2166(s)	31	Fe(II)···CO
			high^d^	2173(l)	38	Fe(III)···CO
				2138	3	physisorbed CO
MIL-100(Fe)^5^	250 °C, 12 h	–173	∼0.0	2170	35	Fe(II)···CO
			low^d^	2185	50	Fe(III)···CO
				2170	35	Fe(II)···CO
			medium^d^	2176	41	Fe(III)···CO
				2166	31	Fe(II)···CO
			high^d^	2170(s)	35	Fe(III)···CO
				2166	31	Fe(II)···CO
				2138	3	physisorbed CO

a The treatment protocol and the temperature (*T*_IR_) and pressure (*P*_IR_) conditions used in the IR measurements are reported. For each frequency, the assignment reported in the original paper is also shown. Reference values for CO in the gas phase in a microporous matrix used for the calculation of the CO stretching frequency shift (Δ*ṽ*_CO_): 2135 cm^–1^ at −173 °C^44,45^ and 2143 cm^–1^ at 25 °C.^45^ Frequencies (cm^–1^), temperatures (°C), pressures (mbar).

b (w) = weak, (s) = shoulder, (l) = large.

c 10% CO in a helium flow.

d Set of spectra recorded at increasing pressures: low and medium pressures are relative values with respect to the highest values used in ref (5) (0.53 mbar, high).

The CO spectra reported in refs (4) and (14) show three bands, whose intensity changes with the treatment temperature: a band at 2189 cm^–1^ is present also after treatments at *T* < 150 °C, associated with CO on Fe(III) sites, and two bands at 2182 (or 2185) and 2173 cm^–1^ gain significant intensity only after the reduction of Fe(III) to Fe(II). The intensity of the signals is slightly different in refs (4) and (14) likely because of a different thermal history of the two samples. In particular, the band at 2173 cm^–1^ is dominant in the spectra of MIL-100-250C reported in ref (4), while in ref (14). the bands at 2173 and 2182 cm^–1^ share the same intensity. Because these bands appear only after the formation of Fe(II) sites and they are not removed after prolonged outgassing at room temperature, they have been associated in refs (4), (5), and (14) with two different Fe(II) sites. In the crystallographic cell of MIL-100, all of the Fe(II) sites are equivalent: the presence of more than one Fe(II) site was explained by the presence of defects in the material. However, no signals typically associated with defects in MOFs^46^ are visible in the spectral regions typical of −OH stretching frequencies and of carboxylate absorption.^5^

The set of spectra recorded at −173 °C is used to follow CO adsorption up to the filling of all of the open iron sites (Table 3).^5^ The description of the bands for the intermediate coverage is the same as that reported at room temperature: the main difference is associated with the position of the bands, shifted of about −6 cm^–1^ as an effect of the temperature.^44,45^ At the highest CO coverage, when all of the open metal sites are coordinated, the CO spectrum is composed of a large single band centered at 2173 cm^–1^ (Δ*ṽ*_CO_ = 38 cm^–1^) for MIL-100(Fe)-150C, while it is shifted to 2166 cm^–1^ (Δ*ṽ*_CO_ = 31 cm^–1^) for MIL-100(Fe)-250C.

The calculations can reproduce the evolution of CO spectra with the coverage and differences observed with different treatment temperatures in the experiments. On the basis of the DFT results, we expect that, with increasing CO pressure in MIL-100(Fe)-150C/200C/250C, the first sites to be occupied by CO molecules will be the Fe(II) sites, followed by the Fe(III) sites in [Fe^II^Fe^III^_2_(μ_3_-O)]^6+^ metal nodes, while Fe(III) in [Fe^III^_3_X(μ_3_-O)]^6+^ will be coordinated only at the highest pressures (Table 2 and Figure 3c,d). The calculated CO adsorption enthalpy of the Fe(III) sites in Fe_3_O-X nodes is less exothermic by 10 kJ mol^–1^ than that for Fe(III) sites in a reduced metal node. Moreover, the adsorption of a second (and of a third) CO on 1CO/Fe_3_O is expected to start before all 1CO/Fe_3_O species are formed because 1CO/Fe_3_O and 2CO/Fe_3_O have similar Δ*H*_CO_^c^ values.

The calculated Δ*ṽ*_CO_ for 1CO complexes is significantly different if the metal node is fully activated (Fe_3_O, +29 cm^–1^) or if it is coordinating the counteranion (Fe_3_O-Cl or Fe_3_O-OH, ∼50 cm^–1^) and/or a water molecule (∼45 cm^–1^). These shifts are close to those observed for the spectra recorded at the lowest coverage on samples degassed at different *T* values (Table 3). In particular, the models are able to reproduce the Δ*ṽ*_CO_ values for both Fe(II)···CO and Fe(III)···CO complexes observed experimentally (35 and 50 cm^–1^, respectively).^5^ The results obtained for Fe_3_O-Cl and Fe_3_O-OH are fully comparable, suggesting that the IR spectra of CO cannot help to distinguish between clusters with different X.

For intermediate coverage, the experimental spectra show the presence of a doublet, where only the relative intensity of the peaks is dependent on *T*, while the position of the peaks is independent. The two peaks have been assigned to Fe(II)···CO (Δ*ṽ*_CO_ = 31 cm^–1^) and Fe(III)···CO (41 cm^–1^).^5^ The calculations suggest an alternative assignment for the higher-frequency band of the doublet. When two CO molecules are adsorbed on the same metal node, each vibrational mode in the CO spectral region is associated with the combination of the modes of the two CO molecules, that is, to the asymmetric or the symmetric stretching of the two CO molecules. For 3CO/Fe_3_O, the three modes are associated with the symmetric stretching of all of the CO molecules (Δ*ṽ*_CO_ = 46 cm^–1^), the asymmetric C–O stretching of the two Fe···CO moieties (47 cm^–1^), and the C–O stretching of Fe···CO (48 cm^–1^), respectively. The predicted shift for modes associated with the 2CO and 3CO complexes is 45 cm^–1^, independent of the metal node. This value is very close to the higher-frequency peak of the doublet (41 cm^–1^) that we assign, based also on the discussion above, to the formation of 2CO complexes on [Fe^II^Fe^III^_2_(μ_3_-O)]^6+^ metal nodes. The band at 31 cm^–1^ is assigned to 1CO complexes on the same nodes (Fe_3_O).

The calculations predict that the shifts on the different Fe_3_O nodes become more and more similar with increasing coverage: all of the IR bands should evolve toward a single band at higher pressures (Figure 3b). Although the predicted shift is slightly higher than the experimental one (48–44 vs 38–35 cm^–1^), the models catch correctly the evolution of the CO spectra with the pressure reported in ref (5). The larger shift obtained in the calculations is associated with a similar description of the three Fe···CO in 3CO/Fe_3_O (Table 2 and Figure 3a). The three CO–Fe distances are very similar, and the three iron sites have similar partial charges, which is not surprising because DFT tends to delocalize the electronic density.

### NO Adsorption

Relevant electronic, structural, and energetic parameters of the NO complexes on Fe_3_O, Fe_3_O-OH, and Fe_3_O-Cl, considering different degrees of hydration, are reported in Tables 4, S3, and S4.

**Table 4:** NO Adsorption on Fe_3_O Clusters Optimized at the UM06-L/def2-TZVP Level in Their Ground Spin State (*S*)^a^

model	2*S* + 1	*d* (Fe–N_NO_)	∠Fe–N–O	Δ*E*_NO_	Δ*E*_NO_^c^	Δ*H*_NO_^c^	Δ*G*_NO_^c^	Δ*ṽ*_NO_
Fe_3_O								
1NO	14	1.802	179	–119.1	–113.7	–108.3	–68.7	–57
2NO	13	1.807	167	–62.2	–58.3	–54.5	–13.8	–71^b^
		2.266	124					–1^b^
3NO	12	1.810	163	–56.3	–52.5	–48.6	–8.8	–82^b^
		2.292	122					–11
		2.284	123					3
Fe_3_O·1H_2_O								
1NO	14	1.809	164	–113.2	–110.7	–105.5	–66.4	–73
2NO	13	1.814	160	–56.1	–52.3	–48.9	–9.9	–85
		2.294	123					–2
Fe_3_O·2H_2_O								
1NO	14	1.817	156	–101.6	–99.2	–93.6	–52.7	–90
Fe_3_O-Cl								
1NO	15	2.270	124	–64.4	–60.4	–56.4	–15.8	5
2NO	16	2.280	123	–27.3	–21.4	–17.5	18.0	5^b^
		2.597	129					24^b^
2NO	14	1.774	169	–5.7	–2.7	0.6	41.1	6
		2.663	126					–32
2NO	12	1.775	178	–28.5	–25.6	–20.2	26.9	5
		2.662	124					–32
Fe_3_O-Cl·1H_2_O								
1NO	15	2.296	123	–58.8	–54.9	–50.6	–9.5	3
Fe_3_O-OH								
1NO	15	2.269	124	–63.4	–59.4	–55.4	–15.2	4
2NO	16	2.277	123	–26.9	–23.1	–19.1	17.3	3^b^
		2.602	129					24^b^
2NO	14	2.670	126	–5.0	0.9	4.1	45.5	–1
		1.775	168					–35
2NO	12	2.316	124	–28.1	–22.2	–17.0	31.3	8
		1.760	177					–7
Fe_3_O-OH·1H_2_O								
1NO	15	2.298	123	–57.1	–53.2	–49.0	–9.6	2

a All of the values reported in this table refer only to the iron sites coordinating a NO molecule. The distance of the reacting iron from the nitrogen of the NO molecule [*d*(Fe–N_NO_) in angstroms] and the Fe···NO angle (∠Fe–N–O, in degrees) are also reported. The stretching frequency shift (Δ*ṽ*_NO_ in reciprocal centimeters) is calculated from the gas-phase values (*ṽ*_NO_ = 1979 cm^–1^). The BSSE-corrected adsorption energy Δ*E*_NO_^c^, adsorption enthalpy Δ*H*_NO_^c^, and adsorption Gibbs free energy Δ*G*_NO_^c^ are reported in kilojoules per mole. The value not corrected for the BSSE is also shown for the energy (Δ*E*_NO_). *H* has been calculated at 1013.25 mbar and 25 °C.

b Vibration involving mainly one NO molecule (made explicit only for 2NO and 3NO complexes).

**Figure 4:**
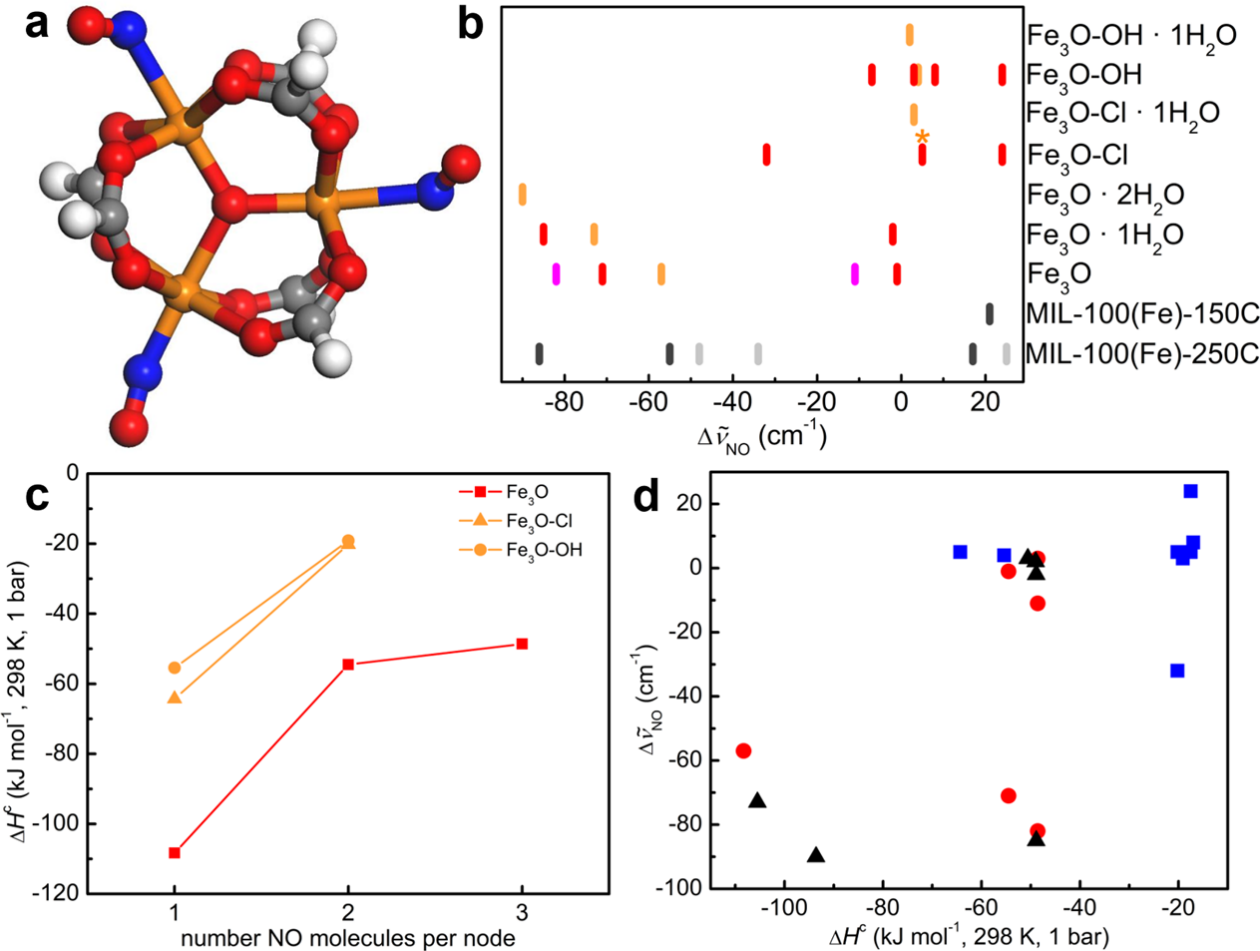
NO complexes on Fe_3_O metal nodes treated at different temperatures. Results were obtained at the UM06-L/def2-TZVP level. (a) Optimized structure of 3NO/Fe_3_O. Color code: red, oxygen; gray, carbon; blue, nitrogen; orange, iron; white, hydrogen. (b) NO vibrational shifts on Fe_3_O, Fe_3_O-OH, and Fe_3_O-Cl for different degrees of hydration of the metal node for the ground-state structures. The colors differentiate different loadings of NO per metal node, and then the NO species can be formed at different pressures. Color code: orange, 1NO complexes; red, 2NO; magenta, 3NO. In all of the complexes, the minimum geometries show the formation of only mononitrosyl species. For the Fe_3_O-Cl/2NO complex, the data shown are for both the 2*S* + 1 = 16 and 12 complexes. The asterisk indicates that this band is also associated with the corresponding 1NO complex (Table 4). The experimental data in ref (24) for MIL-100(Fe)-150C and MIL-100(Fe)-250C at 25 °C (gray lines) and −53 °C (black lines) are also reported. (c) Enthalpy of NO adsorption on Fe_3_O clusters, Δ*H*^c^, as a function of the coverage, as obtained on clusters modeling the fully dehydrated MOFs (treatment temperature ≥150 °C): Fe_3_O, red squares; Fe_3_O-OH, orange circles; and Fe_3_O-Cl, orange triangles. (d) Dependence of the shift of the NO stretching frequency Δ*ṽ*_NO_ on the adsorption enthalpy Δ*H*_NO_^c^ for the clusters modeling Fe_3_O-based materials treated at temperature lower (black triangles) or higher than 150 °C (blue squares, Fe_3_O-Cl; red circles, Fe_3_O). Complexes with lower adsorption enthalpies correspond to species formed at lower equilibrium pressures and higher temperature in the experimental spectra (Table 5).

NO adsorbs on Fe_3_O clusters with a bent geometry in most of the cases (see Figure 4a and ∠Fe–N–O values in Table 4). The calculated enthalpy of NO adsorption, Δ*H*_NO_^c^, is strongly dependent on the NO coverage and on the oxidation state of iron (Table 4 and Figure 4d), unlike CO, for which Δ*H*_CO_^c^ is independent of the coverage and only slightly decreasing with the reduction of the metal node (Table 2 and Figure 3c). Δ*H*_NO_^c^ for 1NO complexes spans a range from −108 to −94 kJ mol^–1^ on the reduced clusters, while for the oxidized cluster, it is halved (−64 kJ mol^–1^ for Fe_3_O-Cl and −51 kJ mol^–1^ for Fe_3_O-Cl·1H_2_O). The adsorption of a second NO is by far less exothermic than the first one, with Δ*H*_NO_^c^ being −50 kJ mol^–1^ for the reduced clusters and −20 kJ mol^–1^ for the oxidized ones. Although no experimental values are reported for the energetics of NO adsorption, Eubank et al.^24^ have studied the competitive adsorption of water in an NO-loaded MIL-100(Fe)-250C(Fe) using IR spectroscopy. They verified by using IR spectroscopy that NO is not fully released at room temperature after switching from a dry to a wet helium flow. Our calculations agree with these observations: the calculated adsorption Gibbs free energy for Fe_3_O is lower than that calculated for water for the first NO (Tables 1 and 4), while it is higher than water for the second NO. The calculations also indicate that Δ*G*_NO_^c^ for Fe_3_O-Cl is always higher than Δ*G*_H_2_O_^c^. These results are an example of the importance of the temperature used in the postsynthetic treatment. For drug delivery, an Fe_3_O-based degassed at ≤150 °C before to be loaded with NO will fully release NO after contact with a water-rich medium, while a MOF treated at ≥150 °C would allow a gradual release in time of NO, with a large amount of NO delivered immediately, followed by a slow desorption due to the gradual substitution of NO by water. MOFs showing a larger affinity for NO than for H_2_O have also been suggested for the environmental removal of NO.^47^ The treatment temperature will have an important effect also on NO capture: Fe_3_O-based MOFs treated at ≤150 °C have Δ*G*_NO_^c^ > Δ*G*_H2O_^c^, and then they will not be able to capture NO in wet streams. The smaller Δ*G*_NO_^c^ than Δ*G*_H_2_O_^c^ of Fe_3_O-based MOFs treated at ≥150 °C would allow one to maintain good selectivity for NO also in wet gas streams, although their capacity will be limited to 1NO molecule per reduced Fe_3_O node.

The calculated Δ*ṽ*_NO_ is strongly dependent on the oxidation state of iron (Table 4 and Figure 4b,d). For a description of the spectra in the region below 800 cm^–1^, see the Supporting Information and Figure S2. For 1NO complexes, Δ*ṽ*_NO_ spans from −57 to −90 cm^–1^ if adsorbed on a reduced cluster, while it is +5 cm^–1^ on oxidized clusters. A vibrational shift smaller than −70 cm^–1^ is in general associated with a bent geometry, that is, with Fe–N–O angles (∠Fe–N–O) significantly different from 180°.^19,50^ The calculations can model this behavior (Figure 4a and Table S3). When the loading (2NO and 3NO complexes) is increased, a different evolution of the bands is predicted for the oxidized and reduced clusters. For the reduced ones, two bands are predicted: one band almost corresponds with the gas-phase value, and a second one is shifted to lower wavenumbers with respect to the 1NO complex. For 2NO/Fe_3_O-Cl, two spin states are calculated to be isoenergetic: 2*S* + 1 = 16 and 12 (Table 4). These spin states have different vibrational shifts, a doublet at 5 and 24 cm^–1^ for 2*S* + 1 = 16 and a doublet at 5 and −32 cm^–1^ for 2*S* + 1 = 12. For the reduced clusters, each vibrational mode is associated mainly with the stretching of a single NO molecule in 2NO complexes (Table 4), unlike for the Fe_3_O-Cl-based clusters, where each mode involves both NO molecules (asymmetric and symmetric stretching modes). In 3NO/Fe_3_O complexes (Figure 4a), the three IR bands are associated with the symmetric and asymmetric stretching of the two NO in Fe(III)···NO (3 and −11 cm^–1^, respectively) and to the N–O stretching in Fe(II)···NO (−82 cm^–1^). This can be explained with the lower similarities of the Fe···NO species in Fe_3_O than in Fe_3_O-Cl clusters because of the presence of Fe(II) species in the former able to engage a stronger interaction with NO species than Fe(III) sites.

Several IR studies reported NO adsorption on Fe_3_O-based MOFs (see refs (14), (24), (48), and (49)). Their results are summarized in Table 5. The experimental Δ*ṽ*_NO_ assigned to NO···Fe(II) complexes in MIL-100(Fe)-250C agrees with the value calculated for 1NO/Fe_3_O: −55 versus −57 cm^–1^, respectively. The shift calculated for the adsorption of the first NO molecule on the oxidized cluster is very small (3–5 cm^–1^), that is, almost indistinguishable from the gas-phase value. The signal of Fe(III)···NO complexes is associated with bands at ∼1895 cm^–1^ (+20 cm^–1^), a shift larger than the one predicted in the calculations. Nevertheless, there is a contradiction between the experimental results reported for IR and volumetric experiments. IR experiments either failed to detect NO adsorption on fully oxidized materials [e.g., MIL-100(Fe)-150C] at room temperature or obtained signals with very small intensity at ∼1895 cm^–1^,^14,24^ a symptom of a small interaction energy of NO with Fe(III) sites. Volumetric measurements indicate a large NO adsorption on the same materials under the same conditions.^24^ Moreover, the volumetric measurements showed that NO can only be partially desorbed, indicating a strong interaction with the material that cannot be explained only by the presence of 2% Fe(II) sites.^24^ The results reported in Table 4 explain this apparent contradiction. The Q band of NO in the gas phase is present in the experimental IR spectrum. The detection of bands slightly shifted from the gas-phase value is then difficult because these bands can be hidden behind the gas-phase absorption. The signals associated with 1NO/Fe_3_O-Cl complexes, if not too intense, can be confused with the gas-phase signals in the experimental spectrum. This can also be the reason why none of the experimental shifts reported in Table 5 are close to zero. This observation can be useful also for the IR characterization of other materials using NO as a probe molecule, in order to avoid that signals associated with Fe(III) sites go unnoticed. The signal at 20 cm^–1^ associated with a general Fe(III)···NO complex is assigned based on the present calculations to the second adsorbed molecule in 2NO/Fe_3_O-Cl clusters, having 2*S* + 1 = 16. The bands observed in the −24 to −34 cm^–1^ range were previously assigned to physisorbed NO^49^ or the Fe(II)···NO complex.^14^ The calculations suggest an alternative assignment, namely, the N–O stretching frequency of the second adsorbed molecule in 2NO/Fe_3_O-Cl clusters, having 2*S* + 1 = 12.

**Table 5:** Review of the Experimental Stretching Frequencies Recorded for NO Adsorbed in Different Fe_3_O-Based MOFs by IR Spectroscopy (*ṽ*_NO_)^a^

material	treatment	*T*_IR_	*P*_IR_	*ṽ*_NO_^b^	Δ*ṽ*_NO_	original assignment
MIL-100(Fe)^24^	150 °C	25	25			
		–53	25	1897	21	Fe(III)···NO
MIL-100(Fe)^24^	250 °C	25	25	1893(w)	17	Fe(III)···NO
				1821	–55	Fe(II)···NO
				1807	–69	Fe(II)···NO
		–53	25	1893	17	Fe(III)···NO
				1821	–55	Fe(II)···NO
				1790	–86	Fe(II)···NO
MIL-127(Fe) or PCN-250^24^	250 °C	25	25	1818	–58	Fe(II)···NO
				1795	–81	Fe(II)···NO
		–53	25	1893	17	Fe(III)···NO
				1818(s)	–58	Fe(II)···NO
				1785(w)	–91	Fe(II)···NO
MIL-100(Fe)^14^	100 °C, 12 h	25	10^c^	1901	25	Fe(III)···NO
	150 °C, 12 h	25	10^c^	1901	25	Fe(III)···NO
				1842(s)	–34	Fe(II)···NO
				1828(w)	–48	Fe(II)···NO
	200/250 °C, 12 h	25	10^c^	1901	25	Fe(III)···NO
				1842	–34	Fe(II)···NO
				1828	–48	Fe(II)···NO
MIL-100(Fe)^48^	250 °C, 12 h	25	unknown	1850(s)	–26	Fe(II)···NO
				1825	–51	Fe(II)···NO
				1813	–63	Fe(II)···NO
Fe(BTC)(Fe)^48^	250 °C, 12 h	25	unknown	1850(s)	–26	Fe(II)···NO
				1813(w)	–63	Fe(II)···NO
MIL-88(Fe)A and MIL-88(Fe)-2OH^49^	150 °C, 3 h	25	1–67	1898	22	Fe(III)···NO
				1813	–63	Fe(II)···NO
			1000	1898	22	Fe(III)···NO
				1845(w)	–31	physisorbed NO
				1813(s)	–63	Fe(II)···NO
MIL-88(Fe)B^49^	80 °C, 3 h	25	1–67	1898	22	Fe(III)···NO
			1000	1898	22	Fe(III)···NO
				1870(s)	–6	Fe(III)···ON
				1852(w)	–24	physisorbed NO
MIL-88(Fe)B-NO_2_^49^	150 °C, 3 h	25	1–1000	1900	24	Fe(III)···NO
				1853(w)	–23	physisorbed NO

a The treatment protocol and the temperature (*T*_IR_) and pressure (*P*_IR_) conditions at which the band appears during the IR measurement are reported. For each frequency, the assignment reported in the original paper is also shown. Reference value for NO in the gas phase: 1876 cm^–1^. Frequencies (cm^–1^), temperatures (°C), and pressures (mbar).

b (w) = weak, (s) = shoulder, (l) = large.

c 1% NO in a helium flow.

No experimental spectra for the full coverage of iron sites by NO molecules have been reported, and they cannot thus be used to benchmark the evolution of the spectra predicted by the calculations. These results suggest that the NO spectra on MOFs treated at ≥150 °C are composed of three families of bands associated with 3NO/Fe_3_O and 2NO/Fe_3_O-Cl complexes (Figure 4b). Unlike CO, characterized by a single broad band at higher CO/Fe coverage, NO can differentiate the different Fe_3_O nodes even at NO/Fe ∼ 1 and is thus a more suitable molecular probe to verify the efficacy of thermal treatments of Fe_3_O-based MOFs.

## 4 Conclusions

Postsynthesis thermal treatments are effective ways to modify the performance of Fe_3_O-based MOFs in most applications. We have used DFT to study the CO and NO adsorption on metal nodes of Fe_3_O-based MOFs, subject to thermal treatments of different efficacy. The calculations allowed us to characterize how the adsorption of small molecules on Fe_3_O-based clusters evolves with the coverage and how the desorption of chemisorbed species (water molecules and counteranions) affects the interaction of the clusters with adsorbates. We compared the simulated IR bands of CO and NO with experimental spectra reported in the literature. The calculations reproduce the changes observed in the spectra. NO showed a larger sensitivity to the presence of adsorbed species than CO at all coverages, and it is then a more suitable molecular probe for quick quality control checks. On the basis of the calculations, we propose to reassign some of the bands previously inaccurately assigned because of the absence of reference data on systems with a structure close to the Fe_3_O structure. Several experimental bands were formerly associated with a large concentration of defects. These bands are here reassigned by considering only crystallographic sites. These findings help in changing the common belief that MILs are highly defective materials and are useful for a more precise assignment of the CO and NO bands in iron-based MOFs, confirming the importance of the synergy between theory and experiments.

The calculated enthalpy of adsorption for both CO and NO was also assessed using the experimental data and compared with the enthalpy of adsorption of water, present often in the different applications of Fe_3_O-based MOFs, as a contaminant or as a solvent. The importance of the interaction of CO and NO with Fe_3_O-based MOFs plays a role in important MOF applications like drug delivery^1,4,11,12,21−23^ and gas mixture purification.^19,4,20^ Future studies should be aimed at enlarging the set of theoretical IR spectra of adsorbates on Fe_3_O-based MOFs, including common probe molecules such as pyridine.

## Supporting Information Available

Additional energetic and electronic parameters for all of the clusters and Cartesian coordinates of all of the optimized structures (PDF)
