# Supplementary material for: Thermal Treatment Effect on CO and NO Adsorption on Fe(II) and Fe(III) Species in Fe3O-Based MIL-Type Metal–Organic Frameworks: A Density Functional Theory Study
Source: Inorg Chem. 2021 Jun 10;60(16):11813–24. doi: 10.1021/acs.inorgchem.1c01044 (PMC8371607; doi:10.1021/acs.inorgchem.1c01044)
Supplement: Supplementary file 1 — ic1c01044_si_001.pdf [file ic1c01044_si_001.pdf]

Supplementary Material

Density Functional Study on the Effect of Thermal Treatment on CO and NO Adsorption on Fe(II) and Fe(III) Species in Fe_3O-Based MIL-Type Metal-Organic Frameworks

Jenny G. Vitillo[1,2]* *and Laura Gagliardi*[3]*

[1]Department of Science and High Technology and INSTM, Università degli Studi dell'Insubria, Via Valleggio 9, I-22100 Como, Italy.

[2]Department of Chemistry, Chemical Theory Center, and Supercomputing Institute, University of Minnesota, 207 Pleasant Street S.E., Minneapolis, Minnesota 55455, United States.

[3]Department of Chemistry, Pritzker School of Molecular Engineering, James Franck Institute, University of Chicago, Chicago, IL 60637, United States.

*E-mail: jg.vitillo@gmail.com

*E-mail: lgagliardi@uchicago.edu

Table of Contents

S1. Additional data on Fe_3O clusters 2
S2. Additional data on CO complexes 3
S3. Additional data on NO complexes 6
S4. Energetic parameters of all the clusters 9
S5. Energetic parameters of the molecular probes 13
S6. Coordinates of M06-L optimized structures 14

S1. Additional data on Fe_3O clusters

Electronic and geometrical parameters calculated for ground state structures of the Fe_3O nodes are reported in Table S1.

Table S1. Fe_3O clusters. All the values reported in this table refer only to the open iron sites. Clusters optimized at the UM06-L/def2-TZVP level in their ground spin state (*S*). Spin densities ρ on the open Fe centers: spin densities are expressed as the difference between the α and β electron densities. CM5 charges *q* on the open Fe centers (in |e|). The distance of the reacting iron from the central tri-iron oxo-centered cluster $d(Fe\text{-}O_c)$ is also reported (Å).

**model**	**ρ(Fe)**			**q(Fe)**			**$d(Fe\text{-}O_c)$**		
Fe_3O	4.02	4.00	4.06	0.87	0.86	0.90	1.870	1.870	1.896
$Fe_3O\cdot 1H_2O$	4.03	4.03		0.87	0.87		1.874	1.878	
$Fe_3O\cdot 2H_2O$	4.04			0.87			1.864		
Fe_3O-Cl	4.14	4.14		0.94	0.94		1.855	1.855	
Fe_3O-Cl$\cdot 1H_2O$	4.13			0.92			1.844		
Fe_3O-OH	4.14	4.14		0.93	0.93		1.852	1.853	
Fe_3O-OH$\cdot 1H_2O$	4.13			0.92			1.843		

S2. Additional data on CO complexes

Additional electronic and geometrical parameters calculated for ground state structures of the CO complexes with the Fe_3O nodes are reported in Table S2.

Table S2. CO adsorption on Fe_3O clusters. All the values reported in this table refer only to the iron sites coordinating a CO molecule. Clusters optimized at the UM06-L/def2-TZVP level in their ground spin state (S). Spin densities ρ on the Fe centers and on the coordinated CO molecule. CM5 charges q on the Fe centers and on the coordinated CO molecule (in |e|). The distance of the reacting iron from the central tri-iron oxo-centered cluster (d(Fe-O_c) in Å) and the Fe···CO angle (∠Fe-C-O, in degrees) are reported. The change of the CO molecular bond (Δd(C-O), in Å) upon adsorption with respect to the gas phase values ($d(CO_{CO})$ = 1.1276 Å) is also shown. Same order as in Table 3.

**model**	**2*S* +1**	**ρ(Fe)**	**ρ(CO)**	***q*(Fe)**	***q*(CO)**	**Δd(C-O)**	**∠Fe-C-O**	**d(Fe-O_c)**
Fe_3O								
1CO	15	3.98	0.03	0.75	0.15	-0.004	178	1.938
2CO	15	4.02	0.04	0.78	0.16	-0.005	178	1.918
		4.02	0.04	0.78	0.16	-0.005	178	1.918
3CO	15	4.03	0.04	0.79	0.16	-0.005	179	1.903
		4.03	0.04	0.79	0.16	-0.005	179	1.903
		4.03	0.04	0.79	0.16	-0.005	180	1.905
$Fe_3O·1H_2O$								
1CO	15	4.01	0.04	0.78	0.16	-0.005	178	1.915
2CO	15	4.03	0.04	0.78	0.15	-0.004	179	1.901
		4.03	0.04	0.78	0.15	-0.004	179	1.901
$Fe_3O·2H_2O$								
1CO	15	4.03	0.04	0.79	0.15	-0.004	180	1.896
Fe_3O-Cl								
1CO	16	4.15	0.04	0.86	0.16	-0.005	180	1.882
2CO	16	4.14	0.04	0.85	0.15	-0.005	180	1.874
		4.14	0.04	0.85	0.15	-0.005	179	1.874

Fe_3O-Cl·1H_2O								
1CO	16	4.14	0.04	0.85	0.14	-0.004	180	1.872
Fe_3O-OH								
1CO	16	4.15	0.04	0.85	0.14	-0.005	180	1.872
2CO	16	4.14	0.04	0.85	0.15	-0.005	179	1.871
		4.14	0.04	0.85	0.15	-0.005	179	1.871
Fe_3O-OH·1H_2O								
1CO	16	4.14	0.04	0.85	0.14	-0.004	179	1.869

Leclerc et al.[1] showed that the MOF spectra undergoes changes upon thermal treatment in vacuum due to the removal of adsorbed species from the metal node in the 700-300 cm^{-1} range. We have reported the calculated spectra in the region below 800 cm^{-1} for the CO complexes with the Fe_3O and Fe_3O-Cl clusters in Figure S1 to verify how CO adsorption affects this spectral region.

The assignment of the bands based on the calculations is reported in Figure S1. It is evident that the largest changes are observed between the Fe_3O (black spectrum) and Fe_3O-Cl clusters (grey spectrum), while CO adsorption slightly affects the spectra. This is due to the low interaction energy of CO CO with the iron centers. As a consequence, the Fe···CO stretching is predicted to be always mixed with other modes involving the iron center.

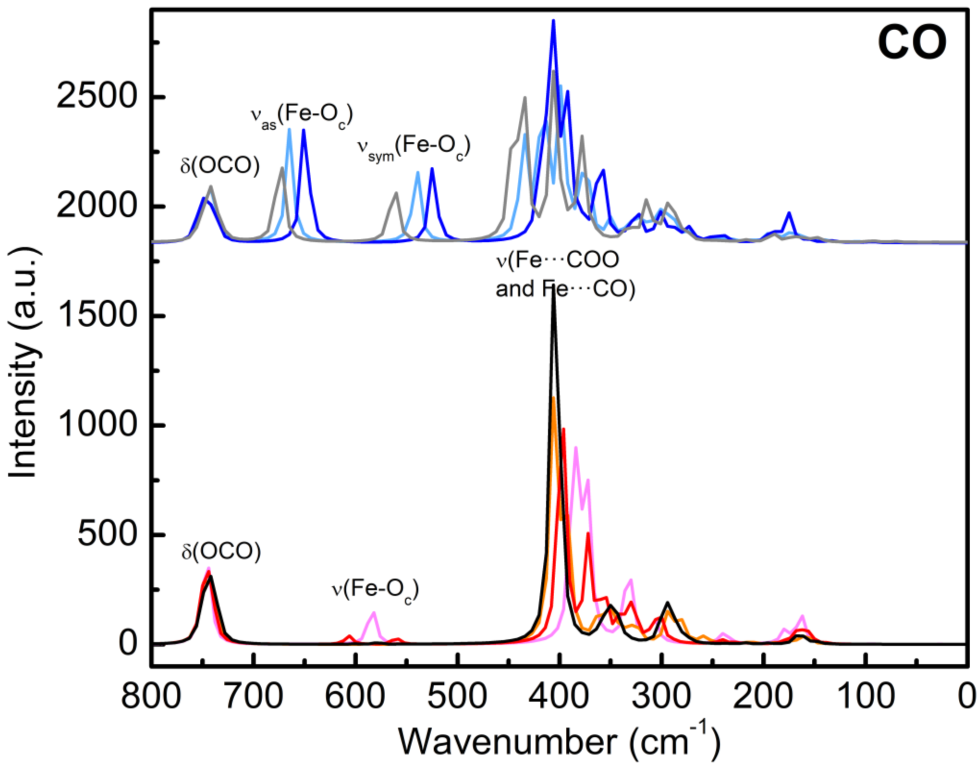

Figure S1. Computed spectra in the region below 800 cm^{-1} of CO complexes on Fe_3O (1CO, orange line; 2CO, red; 3CO, magenta) and Fe_3O-Cl (1CO, light blue line; 2CO, blue line) as obtained at the UM06-L/def2-TZVP level. The spectra of the bare clusters (Fe_3O, black; Fe_3O-Cl, grey) are also added for comparison. O_c is the central oxygen of the metal cluster. IR Peak Half-Width Height = 4 cm^{-1}.

S3. Additional data on NO complexes

Additional electronic and geometrical parameters calculated for ground state structures of the NO complexes with the Fe_3O nodes are reported in Table S3.

Table S3. NO adsorption on Fe_3O clusters. All the values reported in this table refer only to the iron sites coordinating an NO molecule. Clusters optimized at the UM06-L/def2-TZVP level in their ground spin state (S). Spin densities ρ on the Fe centers and on the coordinated NO molecule. CM5 charges q on the Fe centers and on the coordinated NO molecule (in |e|). The distance of the reacting iron from the central tri-iron oxo-centered cluster (d(Fe-O_c) in Å) and the Fe···NO angle (∠Fe-N-O, in degrees) are reported. The change of the NO molecular bond (Δd(N-O), in Å) upon adsorption with respect to the gas phase values ($d(NO_{NO})$ = 1.1471 Å) is also shown. Same order as in Table 5.

**Model**	**2*S* +1**	**ρ(Fe)**	**ρ(NO)**	***q*(Fe)**	***q*(NO)**	**Δd(N-O)**	**∠Fe-N-O**	**d(Fe-O_c)**
Fe_3O								
1NO	14	3.68	-0.99	0.81	0.05	0.005	179	2.081
2NO	13	3.68	-0.99	0.80	-0.11	0.006	167	2.076
		4.01	-0.73	0.86	0.16	-0.015	124	1.881
3NO	12	3.66	-0.99	0.80	-0.12	0.008	163	2.075
		4.00	-0.75	0.85	0.15	-0.014	122	1.864
		4.00	-0.74	0.85	0.15	-0.013	123	1.868
$Fe_3O\cdot 1H_2O$								
1NO	14	3.67	-0.99	0.80	-0.19	0.007	164	2.090
2NO	13	3.67	-0.99	0.80	-0.12	0.008	160	2.088
		4.01	-0.75	0.85	0.15	-0.014	123	1.871
$Fe_3O\cdot 2H_2O$								
1NO	14	3.67	-0.98	0.80	-0.12	0.008	156	2.103
Fe_3O-Cl								
1NO	15	4.01	-0.72	0.86	0.17	-0.016	124	1.885
2NO	16	4.01	-0.73	0.86	0.17	-0.016	123	1.883
		4.17	0.96	0.87	0.09	-0.005	129	1.851

2NO	14	3.11	-0.89	0.85	-0.03	-0.004	169	1.894
		4.01	0.77	0.87	0.23	-0.021	126	1.800
2NO	12	2.80	-0.86	0.88	0.00	-0.004	178	1.823
		3.95	-0.74	0.85	0.16	-0.021	124	1.870
Fe_3O-Cl·1H_2O								
1NO	15	4.01	-0.74	0.85	0.16	-0.015	123	1.876
Fe_3O-OH								
1NO	15	4.01	-0.72	0.86	0.17	-0.016	124	1.882
2NO	16	4.01	-0.73	0.86	0.16	-0.015	123	1.881
		4.17	0.97	0.87	0.09	-0.005	129	1.849
2NO	14	4.02	0.79	0.87	0.21	-0.020	126	1.806
		3.10	-0.90	0.86	-0.03	-0.004	168	1.892
2NO	12	3.96	-0.75	0.85	0.16	-0.015	124	1.870
		2.83	-0.87	0.88	-0.01	-0.007	177	1.828
Fe_3O-OH·1H_2O								
1NO	15	4.01	-0.74	0.85	0.16	-0.015	123	1.872

We report in Figure S2 the calculated spectra in the spectral region below 800 cm^{-1} for the NO complexes on the Fe_3O and Fe_3O-Cl clusters. The assignment of the bands based on the calculations is also reported in Figure S2. Unlike CO, NO interacts strongly with the metal nodes and it perturbs significantly the modes of the metal node (see changes from the bare clusters spectra to spectra for the clusters coordinating NO molecules in Figure S2). Moreover, the Fe···NO stretching is predicted to give rise to a separate peak and to mix only slightly with the node modes.

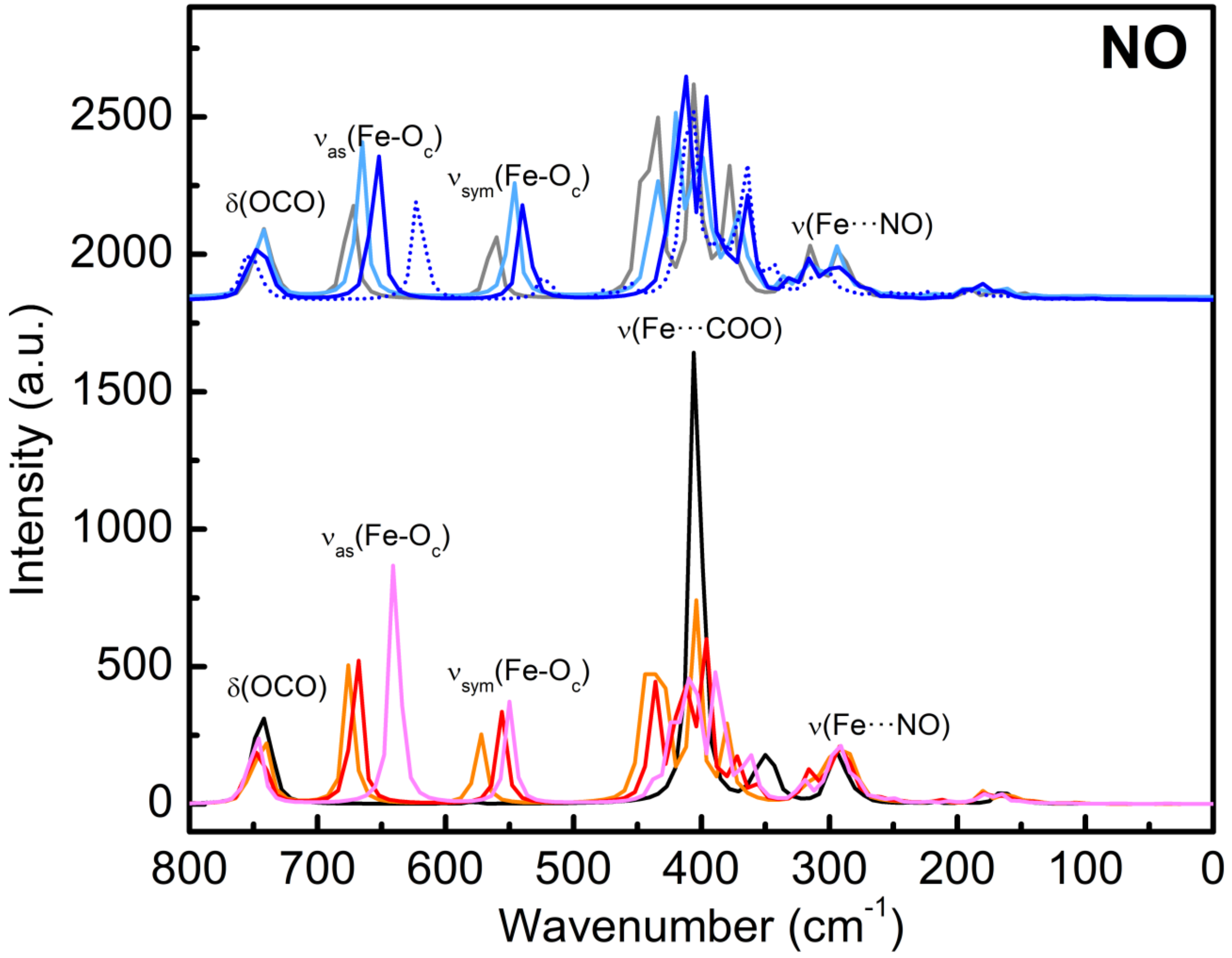

Figure S2. Computed spectra in the region below 800 cm^{-1} of NO complexes on Fe_3O (1NO, orange line; 2NO, red; 3NO, magenta) and Fe_3O-Cl (1NO, light blue line; 2NO, blue line) as obtained at the UM06-L/def2-TZVP level. The spectra of the bare clusters (Fe_3O, black; Fe_3O-Cl, grey) are also added for comparison. O_c is the central oxygen of the metal cluster.

S4. Energetic parameters of all the clusters

In the following tables, the electronic energy, enthalpy and free Gibbs energy for all the complexes are provided.

Table S4. Electronic energy, enthalpy and free Gibbs energy for all the complexes of Fe3O and Fe3O-Cl with NO, as obtained at the UM06-L/def2-TZVPP level in the ground state. These energies are reported as absolute values (E, H^0, G^0, in hartree). H^0 and G^0 have been calculated at 1 atm and 25 °C. The theoretical (S^2_{theo}) and the calculated S^2 ($S(S$+1)) are also reported.

**NO**	$2S$ +1	S^2_{theo}	$S(S$+1)	E	H^0	G^0
Fe_3O						
1NO	14	48.75	48.82	-5132.039506	-5131.854498	-5131.946876
2NO	13	42.00	42.32	-5261.990032	-5261.79575	-5261.89593
3NO	12	35.75	36.72	-5391.938299	-5391.734724	-5391.843011
$Fe_3O·1H_2O$						
1NO	14	48.75	48.815	-5208.51363	-5208.300485	-5208.398176
2NO	13	42	42.3264	-5338.461835	-5338.239591	-5338.345703
$Fe_3O·2H_2O$						
1NO	14	48.75	48.81	-5284.985056	-5284.743824	-5284.847091
Fe_3O-Cl						
1NO	15	56.00	56.01	-5592.253762	-5592.065776	-5592.162218
2NO	16	63.75	63.76	-5722.190991	-5721.993725	-5722.09994
2NO	14	48.75	48.82	-5722.18274778	-5721.985692	-5722.089985
2NO	12	35.75	36.05	-5722.191438	-5721.993609	-5722.095406
Fe_3O-Cl·$1H_2O$						
1NO	15	56	56.01	-5668.726649	-5668.510611	-5668.612809
Fe_3O-OH						
1NO	15	56.00	56.01	-5207.86258818	-5207.662939	-5207.760281
2NO	16	63.75	63.76	-5337.79966766	-5337.590702	-5337.697461
2NO	14	48.75	48.82	-5337.79132949	-5337.582646	-5337.687511
2NO	12	35.75	36.06	-5337.80011657	-5337.590668	-5337.692931
Fe_3O-OH·$1H_2O$						
1NO	15	56	56.01	-5284.33504842	-5284.107284	-5284.210344

Table S5. Electronic energy, enthalpy and free Gibbs energy for all the complexes of Fe3O, Fe3O-Cl, and Fe3O-OH with CO, as obtained at the UM06-L/def2-TZVPP level in the ground state. These energies are reported as absolute values (E, H^0, G^0, in hartree). H^0 and G^0 have been calculated at 1 atm and 25 °C. The theoretical (S^2_{theo}) and the calculated S^2 ($S(S$+1)) are also reported.

**CO**	2S +1	S^2_{theo}	$S(S$+1)	E	H^0	G^0
Fe_3O						
1CO	15	56	56.00	-5115.428892	-5115.243785	-5115.33612
2CO	15	56	56.0003	-5115.42889179	-5228.594149	-5228.694275
3CO	15	56	56.00	-5342.149093	-5341.943866	-5342.051747
$Fe_3O·1H_2O$						
1CO	15	56	56.00	-5191.9045678	-5191.691266	-5191.788613
2CO	15	56	56.00	-5305.2639316	-5305.040573	-5305.145835
$Fe_3O·2H_2O$						
1CO	15	56	56.00	-5268.37858881	-5268.137338	-5268.24095
Fe_3O-Cl						
1CO	16	63.75	63.75	-5575.662575	-5575.473969	-5575.570612
2CO	16	63.75	63.75	-5689.021286	-5688.822701	-5688.927542
Fe_3O-Cl·1H_2O						
1CO	16	63.75	63.75	-5652.136025	-5651.919524	-5652.022383
Fe_3O-OH						
1CO	16	63.75	63.75	-5191.271547	-5191.071245	-5191.168404
2CO	16	63.75	63.75	-5304.630034	-5304.419717	-5304.52492
Fe_3O-OH·1H_2O						
1CO	16	63.75	63.7501	-5267.744699	-5267.516375	-5267.619032

Table S6. Electronic energy, enthalpy and free Gibbs energy for Fe3O, Fe3O-Cl, and Fe3O-OH and all their complexes with H_2O, as obtained at the UM06-L/def2-TZVPP level in the ground state. These energies are reported as absolute values (E, H^0, G^0, in hartree). H^0 and G^0 have been calculated at 1 atm and 25 °C. The theoretical (S^2_{theo}) and the calculated S^2 ($S(S+1)$) are also reported.

**H_2O**	$2S+1$	S^2_{theo}	$S(S+1)$	E	H^0	G^0
Fe_3O	15	56	56.05	-5002.06733658	-5001.892189	-5001.976372
$Fe_3O{\cdot}1H_2O$	15	56	56.00	-5078.54370124	-5078.340369	-5078.429647
$Fe_3O{\cdot}2H_2O$	15	56	56.00	-5155.019517	-5154.788212	-5154.883763
$Fe_3O{\cdot}3H_2O$	15	56	56.00	-5231.493084	-5231.233484	-5231.3337
Fe_3O-Cl	16	63.75	63.75	-5462.30241012	-5462.123779	-5462.21237
Fe_3O-Cl$\cdot 1H_2O$	16	63.75	63.75	-5538.777436	-5538.57083	-5538.665392
Fe_3O-Cl$\cdot 2H_2O$	16	63.75	63.75	-5615.250187	-5615.015538	-5615.115797
Fe_3O-OH	16	63.75	63.75	-5077.911636	-5077.721302	-5077.810663
Fe_3O-OH$\cdot 1H_2O$	16	63.75	63.75	-5154.386466	-5154.168127	-5154.262907
Fe_3O-OH$\cdot 2H_2O$	16	63.75	63.75	-5230.858671	-5230.612036	-5230.71194

The calculated spectra for H_2O in the spectral region typical of the bending mode ($\delta(H_2O)$) of the water molecule (1700-1600 cm^{-1}) are shown in Figure S3. The assignment of the bands based on the calculations is also reported in the plot. It is noteworthy that $\delta(H_2O)$ is shifted, with respect to the gas phase value, to higher wavenumbers after adsorption on the reduced cluster, and to lower wavenumbers for the oxidized cluster.

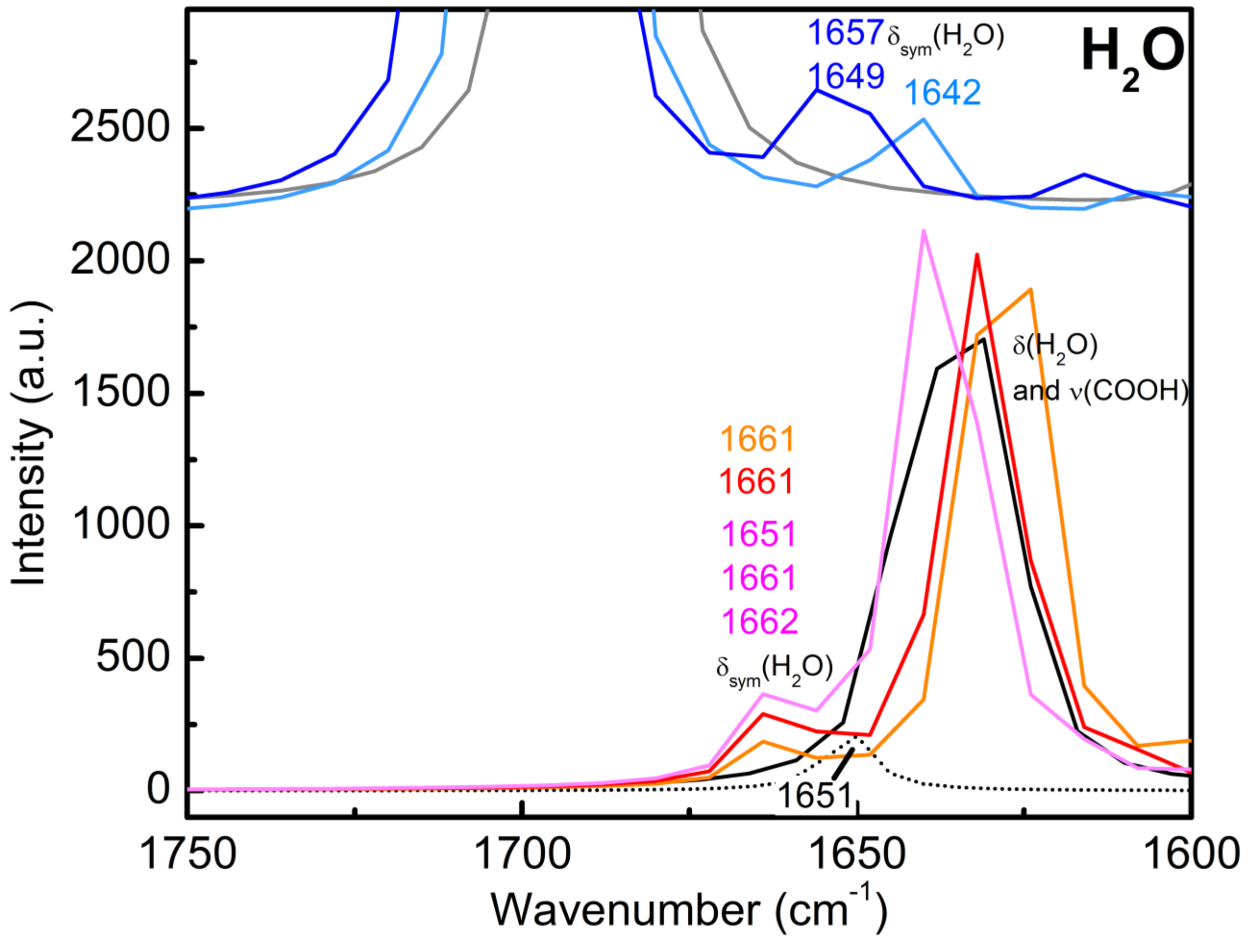

Figure S3. Computed spectra in the bending region of H_2O complexes on Fe_3O ($1H_2O$, orange line; $2H_2O$, red; $3H_2O$, magenta) and Fe_3O-Cl ($1H_2O$, light blue line; $2H_2O$, blue line) as obtained at the UM06-L/def2-TZVP level. The spectra of the bare clusters (Fe_3O, black; Fe_3O-Cl, grey) are also added for comparison.

S5. Energetic parameters of the molecular probes

In the following table, the absolute electronic energy, enthalpy, and free Gibbs energy for the molecular probes used in this study are reported.

Table S7. Electronic energy (E), enthalpy (H^0) and free Gibbs energy (G^0) obtained at the M06-L level for relevant molecules used in this study not reported in the other tables. All energies are reported in hartree. H^0 and G^0 have been calculated at 1 atm and 25 °C. All the energies are in hartree.

Molecule	2S+1	E	H^0	G^0
NO	2	-129.926823134	-129.919010	-129.942302
CO	1	-113.343860719	-113.335540	-113.357966
H_2O	1	-76.4432684687	-76.418120	-76.440195

S6. Coordinates of M06-L optimized structures

The coordinates of all the structures optimized in their ground spin state at the M06-L/def2-TZVP level are reported in the following in the *Gaussian 16* input format (all the values are in Å). They can be read by several freeware software by adding as header this:

```
%nprocshared=24
%mem=17600MB
%chk=MIL-100-NOandCO-M06L.chk
#p opt gfinput m06l test

MIL-100 cluster

0 1
place here the coordinates
add at the end a blank line
```

and saving the file with a .gjf extension.

Fe_3O, 2*S* +1 = 15

```
O,0,-1.3965305359,-2.1550536682,1.4974634092
O,0,-1.4249299478,-0.1579202181,2.556757216
C,0,-1.8056631582,-1.3333577329,2.3547705908
H,0,-2.6111398236,-1.6982873768,3.0151725716
O,0,1.4221694331,-0.1727766066,-2.448307975
O,0,1.4042432915,-2.1654426771,-1.3873753755
C,0,1.8123275327,-1.345288486,-2.2465291636
H,0,2.6213369135,-1.706173551,-2.9040907075
O,0,-1.428899418,2.3436257061,1.186473538
O,0,-1.4035628581,-2.1653071246,-1.387572278
O,0,-1.4229986023,-0.1723629226,-2.4479649671
C,0,-1.8125004882,-1.3451059014,-2.2462794138
H,0,-2.6216993812,-1.7061952744,-2.9034966237
O,0,1.4251123706,-0.1571948062,2.5566937524
O,0,1.3973820948,-2.1543055425,1.4973606689
C,0,1.8062219038,-1.332524996,2.35469992
H,0,2.6118131232,-1.6971842475,3.015110605
Fe,0,-0.0001429864,0.9716426461,1.624782043
O,0,1.4281985486,2.3440500024,1.1860260511
Fe,0,-0.0001364224,0.952927878,-1.5228728885
O,0,1.4235951069,2.3301281247,-1.0701033539
O,0,-1.4235039451,2.3304567541,-1.0696569978
Fe,0,0.0002549988,-1.941619777,0.0458885284
O,0,-0.0001521382,-0.0719149875,0.0721478962
C,0,1.8152202742,2.7215151476,0.0544222441
H,0,2.6040572149,3.4926252688,0.0488314748
C,0,-1.8155689524,2.7214250354,0.0548464944
H,0,-2.6044890274,3.4924490719,0.0492507598
```

Fe_3O/1NO, 2*S* +1 = 14

```
O,0,-2.6338036986,0.9799889874,1.3369871444
O,0,-2.5517824145,-1.2701339625,1.4212564693
C,0,-2.9832494799,-0.1423348528,1.765086099
H,0,-3.7644882391,-0.1448382608,2.5434181746
O,0,1.7391356603,1.3670373568,-1.6130380681
O,0,-0.1184728161,2.607985349,-1.2998401641
C,0,0.9568343134,2.2898576545,-1.8853714893
H,0,1.2178035665,2.9299181819,-2.7466394982
O,0,-0.1103917298,-2.6047715689,1.3104239342
O,0,-0.3270340914,2.5721368043,1.5151816225
O,0,1.7248347717,1.6424241577,1.3564350631
C,0,0.8865284577,2.4130432314,1.8456068568
H,0,1.218374425,3.0555556845,2.6800472031
O,0,-2.6340288211,-0.985442793,-1.3257869646
O,0,-2.5556320856,1.2648624707,-1.4102492026
C,0,-2.9858098599,0.136322941,-1.7533697614
H,0,-3.7681278008,0.1375890632,-2.5306428107
Fe,0,-1.165715491,-1.5813199768,-0.0205533805
O,0,-0.3272662818,-2.5773866504,-1.5049571578
Fe,0,1.8793493485,0.0018896505,-0.0033518158
O,0,1.7215817708,-1.637865487,-1.3647024015
O,0,1.7506873813,-1.3644840763,1.6056810471
Fe,0,-1.1684459611,1.578451212,0.0302673012
O,0,-0.2012344509,-0.0005913277,0.0018284988
C,0,0.8829537717,-2.4134708346,-1.8452593574
H,0,1.2110846009,-3.0565227919,-2.6807542974
C,0,0.9696655766,-2.2857721034,1.8866734115
H,0,1.2366552512,-2.9235041274,2.7478303831
N,0,3.6816454964,0.0032388909,-0.0155904978
O,0,4.8331598299,0.0011571779,-0.0508733414
```

Fe_3O/2NO, 2*S* +1 = 13

```
O,0,1.4752339033,2.3892540109,-1.4223771324
O,0,2.3643751827,0.3499671464,-1.7954957511
C,0,2.168022467,1.546636509,-2.0600000707
H,0,2.658656874,1.9440096005,-2.9665129351
O,0,-2.4352550613,0.3963607559,1.6164781877
O,0,-1.3345758894,2.3542356236,1.4258120419
C,0,-2.1861808798,1.590172767,1.9244535981
H,0,-2.7986205596,2.00850162,2.741676613
O,0,0.8487326296,-2.2229147125,-1.3384489869
O,0,-1.3042488438,2.513073095,-1.3423618928
O,0,-2.5174437662,0.6193020065,-1.1812460337
C,0,-2.2605059257,1.7554959958,-1.6360498269
H,0,-2.9614734785,2.1459026398,-2.3941219263
O,0,2.7392712766,0.682211733,1.1490449052
O,0,1.4587091648,2.5244505134,1.3977631942
C,0,2.4495380103,1.7786193991,1.6517118532
H,0,3.1335697832,2.1737242639,2.423613756
Fe,0,1.907094872,-0.8298156503,-0.1077788924
O,0,1.2617696529,-1.8745884617,1.6131245355
Fe,0,-1.5331889806,-0.6796025822,0.1307403874
O,0,-0.9825584373,-2.0456379605,1.4661217738
O,0,-1.3966143679,-1.9881118125,-1.3756741914
Fe,0,0.1287440866,2.0517176981,0.0067581013
O,0,0.1161290456,0.215096516,0.001250435
C,0,0.1478033168,-2.2709556423,1.9861344136
H,0,0.121478635,-2.9096879152,2.8872521068
C,0,-0.2876731057,-2.4687745167,-1.7619492523
H,0,-0.3727654487,-3.201573797,-2.5844381856
N,0,-3.6009565314,-1.6053175312,0.1608678072
O,0,-4.435624447,-1.3044248242,-0.5420820775
N,0,3.4583726613,-1.7553221342,-0.1441269279
O,0,4.4318511613,-2.3315443534,0.0805973733
```

Fe_3O/3NO, 2*S* +1 = 12

```
O,0,1.4358987409,-2.0990713124,-1.2898072952
O,0,-0.7881601274,-2.4506446989,-1.4091761397
C,0,0.405978195,-2.6330718726,-1.7517297119
H,0,0.5603414724,-3.3608634263,-2.5681830627
O,0,0.8629408996,2.2219034284,1.7594940429
O,0,2.475163217,0.6683053616,1.487471475
C,0,1.9090724094,1.634228265,2.0735179778
H,0,2.429447561,1.9933502797,2.9798759854
O,0,-2.551621751,-0.2536407927,-1.3176899687
O,0,2.6282111401,0.4703792832,-1.3778311729
O,0,1.3330729177,2.3103825093,-1.2031738829
C,0,2.2561029765,1.6413942311,-1.6868840702
H,0,2.8466098207,2.1093645639,-2.4952475945
O,0,-0.5864636922,-2.5020423523,1.4109874522
O,0,1.6172500314,-2.0400349296,1.5200197867
C,0,0.5719952297,-2.6506302837,1.8541082173
H,0,0.6969391289,-3.4145102775,2.6414237336
Fe,0,-1.4102144011,-1.1695567904,0.0712782815
O,0,-2.5936904765,-0.4806009073,1.530812785
Fe,0,-0.3808867436,2.0959996511,0.0612213643
O,0,-2.0557079579,1.6995471928,1.3125072351
O,0,-1.5727720485,1.7542409305,-1.6461802976
Fe,0,1.7548802176,-0.5696404115,0.0991259053
O,0,-0.0046832518,0.0551900833,0.0496099928
C,0,-2.6738551329,0.7512757822,1.8152837065
H,0,-3.3959910964,0.9892585688,2.6171106826
C,0,-2.3515743356,0.8320488815,-1.9318377192
H,0,-2.9656493574,0.9663276144,-2.8414066041
N,0,-0.6436134606,3.8847300076,-0.0189981645
O,0,-0.5785814834,4.9971381863,-0.3221569985
N,0,3.8467287675,-1.4835865105,0.0332170159
N,0,-3.1157153034,-2.6979496904,-0.0289762707
O,0,4.1610828283,-2.2724716345,-0.7170987078
O,0,-3.9540939337,-2.62257293,-0.7886169788
```

Fe_3O/1CO, 2*S* +1 = 15

```
O,0,-2.6622495653,1.1463374106,1.4075450861
O,0,-2.6057674475,-1.11201494,1.4245778507
C,0,-3.0092358927,0.0087918362,1.80911445
H,0,-3.7685555344,-0.0015565972,2.6101439352
O,0,1.7066124638,1.4353361078,-1.4626588265
O,0,-0.2185588004,2.6102331672,-1.4077696469
C,0,0.927598794,2.3353823579,-1.8443305644
H,0,1.2847018917,2.9733140668,-2.6713472572
O,0,-0.1973612417,-2.6093647835,1.4282567973
O,0,-0.2062871955,2.6083114972,1.4147461182
O,0,1.7181571436,1.4316198758,1.4535287485
C,0,0.942875441,2.3318764847,1.8422625165
H,0,1.3068168748,2.9683890015,2.6673945865
O,0,-2.6121148239,-1.1094804189,-1.4154876631
O,0,-2.6749954921,1.1484956767,-1.3808852574
C,0,-3.0216632033,0.013021443,-1.7885122818
H,0,-3.7869476823,0.0064799644,-2.5838898002
Fe,0,-1.2247052478,-1.6140794123,0.0009270437
O,0,-0.2051551372,-2.6073083022,-1.4331607949
Fe,0,1.5790837297,-0.0339203167,-0.0059056257
O,0,1.7483115761,-1.4806140607,-1.4708112534
O,0,1.7577524357,-1.4849437321,1.4534710253
Fe,0,-1.2822261824,1.6557195207,0.0074257137
O,0,-0.357054512,0.0396468602,0.0018646679
C,0,0.9546871465,-2.3704254964,-1.8503952115
H,0,1.3151599619,-3.0324404562,-2.6568021125
C,0,0.9655483637,-2.3740365839,1.8377721226
H,0,1.3306862265,-3.0369730227,2.6413198073
C,0,3.8942566105,0.0386587166,-0.0141807351
O,0,5.0154402988,0.1085641352,-0.018470439
```

Fe_3O/2CO, 2*S* +1 = 15

```
O,0,-2.6645554514,-1.0283725424,-1.428801597
O,0,-2.5599668273,1.223863137,-1.4196221236
C,0,-2.9967181485,0.1165184148,-1.8131162244
H,0,-3.7689987944,0.1591852126,-2.6008499566
O,0,1.7928698187,-1.4884962345,1.4417192732
O,0,-0.1876321621,-2.5620677836,1.440043359
C,0,0.9776253806,-2.34869032,1.8485304781
H,0,1.328890132,-2.9980392832,2.6695278168
O,0,-0.0840467565,2.5646963153,-1.4217198533
O,0,-0.1871263137,-2.5634608236,-1.4392749178
O,0,1.7940564554,-1.4912038784,-1.4375892228
C,0,0.9790466614,-2.3511457398,-1.845540224
H,0,1.331582224,-3.00122227,-2.6654115219
O,0,-2.5586973582,1.2265642734,1.4191803118
O,0,-2.6657483389,-1.0255885091,1.4269896861
C,0,-2.9965809313,0.119387727,1.812054391
H,0,-3.7687345082,0.1624996422,2.5999011107
Fe,0,-1.1578331796,1.5934837383,-0.0010368988
O,0,-0.0831452788,2.5675816289,1.4170406528
Fe,0,1.6448291585,-0.0450228727,0.0005691582
O,0,1.8609455558,1.4256511204,1.4270410973
O,0,1.8603654773,1.4232499204,-1.4288935819
Fe,0,-1.3162669083,-1.6490700956,-0.0002715813
O,0,-0.2732013259,-0.0394211157,0.0001326614
C,0,1.0830512607,2.3292505121,1.8107092135
H,0,1.4689213508,3.0001686832,2.5979435044
C,0,1.0822989783,2.3257764014,-1.8146749653
H,0,1.468105082,2.9950967314,-2.6033067697
C,0,4.0293082666,-0.1076713862,-0.0002392798
O,0,5.149203768,-0.1858478932,-0.0010614847
C,0,-2.5663175389,-3.6804419697,0.003391719
O,0,-3.1125293784,-4.6612190208,0.0059700099
```

Fe_3O/3CO, 2*S* +1 = 15

```
O,0,-2.3655467906,-1.0292537067,-1.4347240213
O,0,-2.2575148287,1.2203426582,-1.4389198823
C,0,-2.6896338142,0.1131889521,-1.8340836817
H,0,-3.449095183,0.1476495694,-2.6351993084
O,0,2.0666506707,-1.5193649205,1.4422010606
O,0,0.0805654591,-2.5812404319,1.4311789114
C,0,1.2503220582,-2.3843315923,1.8348603817
H,0,1.6040422815,-3.0546125821,2.6381487325
O,0,0.2963786002,2.5719161233,-1.4360461441
O,0,0.08060264,-2.5811344526,-1.433963296
O,0,2.067925108,-1.5215586347,-1.4391249911
C,0,1.2516922583,-2.3854667815,-1.8343511724
H,0,1.6068469217,-3.055911106,-2.636860665
O,0,-2.2551230583,1.2200430451,1.4434309525
O,0,-2.3672192796,-1.0293608565,1.4304252175
C,0,-2.6890984774,0.1121320608,1.8343564476
H,0,-3.4482415512,0.1448232216,2.6358584768
Fe,0,-0.8710199934,1.6883583415,0.0012917277
O,0,0.297209454,2.5729246353,1.436808579
Fe,0,1.9008648904,-0.0687711194,0.000423713
O,0,2.1996004196,1.3665496687,1.4373888466
O,0,2.1982430042,1.364673893,-1.4388745876
Fe,0,-1.0208200511,-1.6141383069,-0.0016177356
O,0,-0.0013060556,-0.004535675,0.0003169428
C,0,1.4518972237,2.2909015813,1.8320850598
H,0,1.8606151022,2.9361169071,2.6297968851
C,0,1.4503427453,2.2891476839,-1.8329100659
H,0,1.8581339655,2.9337372827,-2.6316102394
C,0,4.3058670925,-0.1976439999,-0.0003775953
O,0,5.4261047875,-0.2741168985,-0.0009459422
C,0,-2.0210179883,3.8085369878,-0.0007524739
C,0,-2.3203163033,-3.6467156939,0.0006267131
O,0,-2.5701687585,4.7879338116,-0.0020808298
O,0,-2.9295525495,-4.5898396649,0.0030669843
```

$Fe_3O \cdot 1H_2O$, 2*S* +1 = 5

```
O,0,-2.5264573197,-1.1205537163,-1.4599815949
O,0,-2.5522739571,1.1334685006,-1.4556716137
C,0,-2.914079214,0.0028283664,-1.8582970046
H,0,-3.6632439714,-0.0041327793,-2.6680135271
O,0,1.7849430155,-1.4354120846,1.6159883634
O,0,-0.1702409858,-2.5465301874,1.4467038545
C,0,0.9683464462,-2.3338579318,1.9265641197
H,0,1.2897483657,-3.0233591256,2.7257651415
O,0,-0.1030060033,2.564005413,-1.3854273546
O,0,-0.0602660001,-2.5129495408,-1.3949214285
O,0,1.928309111,-1.4616568147,-1.2338042248
C,0,1.1363652893,-2.3129829449,-1.7046677258
H,0,1.541616368,-2.9696159791,-2.4948786586
O,0,-2.6563323844,1.1221188724,1.3639783099
O,0,-2.6310786768,-1.1322781042,1.3627683771
C,0,-3.0468212561,-0.0097204883,1.7349714241
H,0,-3.8537919154,-0.0192862855,2.4871908281
Fe,0,-1.219284583,1.596378405,0.004717212
O,0,-0.227462527,2.5944913307,1.4548256197
Fe,0,1.720499317,0.0446398758,0.2342145667
O,0,1.7509153852,1.5246385725,1.6156204062
O,0,1.903521235,1.5466718086,-1.2323045457
Fe,0,-1.1844948057,-1.5626542136,0.000063263
O,0,-0.1911225296,0.0251530521,0.0485312
C,0,0.9159239962,2.403647265,1.932713305
H,0,1.2232144759,3.0948200912,2.7359138363
C,0,1.0949603307,2.3839172753,-1.7010201754
H,0,1.4857731811,3.0455367423,-2.4942390022
O,0,3.8607818375,0.0541493505,-0.119969267
H,0,3.9164688287,0.821605266,-0.7085345287
H,0,3.9201723063,-0.7108170414,-0.7117132755
```

$Fe_3O \cdot 1H_2O/1NO$, 2*S* +1 = 14

```
O,0,-2.6151451413,-1.0673164686,-1.3767130072
O,0,-2.5241442091,1.173952067,-1.5886480365
C,0,-2.9419427372,0.0285649126,-1.8830685216
H,0,-3.6928568865,-0.016134848,-2.6906368246
O,0,1.7099455739,-1.285695562,1.6574395947
O,0,-0.1475149286,-2.5298026637,1.365526278
C,0,0.9238534808,-2.2003506399,1.9487706063
H,0,1.179364656,-2.8180330672,2.8281591913
O,0,-0.0229676141,2.6142755763,-1.4401244492
O,0,-0.2907325839,-2.5889638317,-1.448798281
O,0,1.7561303417,-1.651139891,-1.3039055896
C,0,0.9241091231,-2.4451911285,-1.769640406
H,0,1.271291931,-3.1230550871,-2.5694281525
O,0,-2.6995253413,1.053213579,1.223033817
O,0,-2.5688271882,-1.188986341,1.392004276
C,0,-3.0290291644,-0.0618078779,1.6874756261
H,0,-3.82281299,-0.0570552964,2.4550490395
Fe,0,-1.1909674734,1.7000359269,-0.1372802997
O,0,-0.282709148,2.6236841346,1.4376314479
Fe,0,1.865357904,0.0224930526,0.0092769763
O,0,1.7602187092,1.6741195112,1.3313854765
O,0,1.808899216,1.317969489,-1.6654481761
Fe,0,-1.1467343418,-1.5085300482,-0.0199660819
O,0,-0.2230160165,0.0836862743,-0.049881818
C,0,0.9147079698,2.4433685258,1.8069626307
H,0,1.2307801908,3.0665664796,2.6635855135
C,0,1.0511767837,2.2406027705,-1.9955148369
H,0,1.3362148911,2.8295393355,-2.885577605
N,0,3.6725725836,-0.0361602031,-0.0447021272
O,0,4.7657382153,-0.1920941832,-0.3790841655
O,0,-2.4303037494,3.5065212265,0.1051988919
H,0,-1.9621433711,3.9115719805,0.8496765586
H,0,-3.2317988353,3.1327705557,0.4965043542
```

$Fe_3O\cdot 1H_2O/2NO$, $2S+1 = 13$

```
O,0,-2.5466648892,-0.1708675803,-1.3225777641
O,0,-1.6856835714,1.8948975038,-1.5914841378
C,0,-2.4579609602,0.9490798805,-1.8722995694
H,0,-3.1514715115,1.1309622945,-2.7125860108
O,0,1.4657802819,-1.7132055063,1.8052153043
O,0,-0.7046060289,-2.2825383034,1.5862314938
C,0,0.4208765189,-2.2791748372,2.1600176283
H,0,0.464245224,-2.864983549,3.0960807125
O,0,1.1594815637,2.3747841576,-1.4426282897
O,0,-0.9320891189,-2.4742541412,-1.2729170955
O,0,1.3100483419,-2.2654804835,-1.1438497256
C,0,0.2620131768,-2.756385507,-1.5853876525
H,0,0.3605867634,-3.5452801255,-2.3527020083
O,0,-1.9025005218,1.9621259783,1.2355313651
O,0,-2.5821536044,-0.1695942713,1.4979717767
C,0,-2.6047304884,1.060704229,1.7417824238
H,0,-3.3397840937,1.3809429329,2.5019475091
Fe,0,-0.2644742422,1.9400829615,-0.1310193561
O,0,0.9103691011,2.5640199278,1.4274865101
Fe,0,1.9961225251,-0.6462653284,0.066674355
O,0,2.5269981788,1.0011760034,1.276205241
O,0,2.3565783786,0.4791799983,-1.6871298618
Fe,0,-1.3780336389,-1.1030047263,0.1232089282
O,0,0.0479827617,0.1029148229,0.0085927835
C,0,2.0021630928,2.0166795917,1.7545934651
H,0,2.549846595,2.524870024,2.5695996951
C,0,1.9922089741,1.6172559372,-2.0178802433
H,0,2.4497989927,2.0426470825,-2.929328941
N,0,3.6652474055,-1.3520736623,-0.0045949631
O,0,4.5656869213,-1.9718697518,-0.3775548809
O,0,-0.7636890834,4.1030616917,0.0199435209
H,0,-0.1542038574,4.3310277414,0.7368657964
H,0,-1.6291584173,4.0522079932,0.4479170837
N,0,-3.2325372554,-2.4533886727,0.1588593524
O,0,-4.0852385141,-2.3588073061,-0.5812384452
```

$Fe_3O\cdot 1H_2O/1CO$, $2S+1 = 15$

```
O,0,-2.659370363,-1.1966982548,-1.4160170991
O,0,-2.6778553415,1.05040671,-1.5954801885
C,0,-3.0563673356,-0.1063757123,-1.8909496688
H,0,-3.8349862345,-0.1767931914,-2.6698323648
O,0,1.7301995043,-1.4268559513,1.48013897
O,0,-0.225765688,-2.5441882545,1.4371896725
C,0,0.9323447139,-2.3151853909,1.8580485006
H,0,1.2896555472,-2.9781051153,2.6650699762
O,0,-0.1291478116,2.5252971301,-1.6389385446
O,0,-0.2068543735,-2.5943999485,-1.3923129466
O,0,1.7699422693,-1.5119342584,-1.3926409134
C,0,0.9719401736,-2.3990248085,-1.7723241342
H,0,1.3491580951,-3.0959098887,-2.540993568
O,0,-2.7353742453,1.1522329428,1.2573778245
O,0,-2.6330780465,-1.0932386209,1.4193422878
C,0,-3.066106147,0.0391757655,1.7309677095
H,0,-3.8337826025,0.0561732007,2.5251285281
Fe,0,-1.3332426187,1.7205336969,-0.2274002694
O,0,-0.1639398021,2.6807209763,1.2251283727
Fe,0,1.5567936154,-0.0057857944,0.0040611967
O,0,1.7242207651,1.467598861,1.4276820557
O,0,1.8146980279,1.3988450428,-1.4655976801
Fe,0,-1.2541289188,-1.5510408006,-0.0021368492
O,0,-0.3556870563,0.0801878756,-0.0504690761
C,0,0.9532908314,2.4053346879,1.7269161796
H,0,1.2983411593,3.0756821699,2.5347316697
C,0,1.053616475,2.2636745729,-1.958623566
H,0,1.4763324353,2.8707601391,-2.7781047386
C,0,3.9400096267,0.0120335156,0.0181029924
O,0,5.0615884751,0.0613891486,0.0035797926
O,0,-2.4505427276,3.5645719891,0.1018542913
H,0,-1.8251092055,4.0093154068,0.6927204082
H,0,-3.1336033466,3.2142204191,0.6930430892
```

$Fe_3O{\cdot}1H_2O/2CO$, $2S+1 = 15$

```
O,0,-2.0001372946,1.9996786019,1.4087510428
O,0,-2.6715426772,-0.1477772245,1.5127583699
C,0,-2.6946353492,1.0567506517,1.854550053
H,0,-3.4155460559,1.3193701317,2.6485548406
O,0,2.3410858165,0.9506912076,-1.4740462552
O,0,0.8169890656,2.6080148924,-1.4650873497
C,0,1.856074364,2.0362967799,-1.867247474
H,0,2.4123016406,2.5520579994,-2.6700413178
O,0,-0.676661277,-2.2889950784,1.5293609349
O,0,0.8186626909,2.6243250953,1.4144563485
O,0,2.3564218398,0.9786983331,1.4060033886
C,0,1.8685813811,2.0620034772,1.8023052602
H,0,2.4331338211,2.5856419139,2.5942455092
O,0,-2.7185891113,-0.1830469433,-1.3567050201
O,0,-1.9791472648,1.9407805289,-1.4806335922
C,0,-2.7056453738,0.9817099303,-1.822113881
H,0,-3.4244164098,1.1864331124,-2.6362219472
Fe,0,-1.5499523372,-1.1132877048,0.1270032914
O,0,-0.704199133,-2.3643543354,-1.3394660204
Fe,0,1.6781841098,-0.340107117,-0.0208882037
O,0,1.4684466587,-1.7856561039,-1.4670760347
O,0,1.5166411282,-1.7871537881,1.421972355
Fe,0,-0.5339254586,2.0463626337,-0.0227103607
O,0,-0.148317685,0.1853719747,0.0054978754
C,0,0.4588353669,-2.4426683375,-1.8030058838
H,0,0.6105463088,-3.1831648098,-2.6091688539
C,0,0.5220710335,-2.4015429296,1.8734554775
H,0,0.7273565306,-3.1319920764,2.6756348757
C,0,3.9825260082,-1.0673328789,-0.0142727481
O,0,5.0491757773,-1.4182407679,0.0077809207
O,0,-3.138097208,-2.5862867397,-0.2171579724
H,0,-2.6501335998,-3.1745483659,-0.8121867153
H,0,-3.6941222692,-2.0563029216,-0.8069733993
C,0,-1.085311153,4.3986452329,-0.0336898468
O,0,-1.356284684,5.4885303355,-0.024556617
```

$Fe_3O{\cdot}2H_2O$, $2S+1 = 15$

```
O,0,2.4346333489,1.3144919318,-1.4055376807
O,0,2.4755059009,-0.925751686,-1.6393007387
C,0,2.8080307675,0.2417388907,-1.9391178959
H,0,3.5194832086,0.3394295762,-2.7785027388
O,0,-2.0015914558,1.2661300803,1.5462918145
O,0,-0.1068551631,2.4840087223,1.6104182169
C,0,-1.2625365926,2.1799146798,1.9838634644
H,0,-1.6841344953,2.788415081,2.8023085282
O,0,0.0037686699,-2.5008233936,-1.5631020286
O,0,-0.2372940009,2.622849224,-1.250971543
O,0,-2.0900734517,1.3419003546,-1.2752474803
C,0,-1.3897774582,2.3145936877,-1.6368974946
H,0,-1.8460812297,2.9794561173,-2.391678523
O,0,2.6620216279,-1.0765505665,1.235202545
O,0,2.5049404225,1.1581220783,1.4724649961
C,0,2.972993531,0.0298100546,1.7394143515
H,0,3.7580584384,0.0053852553,2.5161656323
Fe,0,1.2311562171,-1.6254967092,-0.1964518931
O,0,0.1274943325,-2.6403173177,1.2979357792
Fe,0,-1.6162636501,-0.0851323864,0.0898824823
O,0,-1.8328064843,-1.5463341289,1.4836337763
O,0,-1.9888076791,-1.4736569956,-1.3347384722
Fe,0,1.0816975994,1.7281181903,0.1422080416
O,0,0.2435369178,0.0065384143,0.0108795055
C,0,-1.0134704742,-2.445364405,1.7799622115
H,0,-1.3384646663,-3.1500816296,2.5658944805
C,0,-1.2034677198,-2.3127727851,-1.8365357081
H,0,-1.6310239846,-2.9631644561,-2.6188908548
O,0,2.365048347,-3.4758985514,0.0563384586
H,0,1.7480848671,-3.9392849887,0.6421988336
H,0,3.0509963199,-3.1365876582,0.6503188123
O,0,2.003258913,3.6791300271,-0.197746034
H,0,1.2977116224,4.0776936086,-0.7287214548
H,0,2.668399454,3.4075566839,-0.8475353897
```

$Fe_3O \cdot 2H_2O$/1NO, 2*S* +1 = 14

```
O,0,-2.0091196355,2.0461121976,1.2770583585
O,0,-2.5755075911,-0.1116953727,1.5799329157
C,0,-2.6239997577,1.1110697914,1.8393455609
H,0,-3.2961197656,1.3993469716,2.6675068521
O,0,2.1780145197,0.7496205162,-1.8113545613
O,0,0.8776835432,2.5704022627,-1.5416956402
C,0,1.7516330797,1.8698768827,-2.1270728647
H,0,2.1841836242,2.3332530169,-3.0320689163
O,0,-0.7052330083,-2.2872679329,1.3993141552
O,0,0.7881999433,2.6710582366,1.3394752837
O,0,2.3936228648,1.0971610907,1.182503781
C,0,1.873931148,2.1125052555,1.6662278317
H,0,2.421791725,2.6087524166,2.4883945339
O,0,-2.8134505704,-0.0374150429,-1.250621554
O,0,-1.9408395283,2.01385901,-1.5639723193
C,0,-2.7507903508,1.0893862563,-1.7943373834
H,0,-3.5058563845,1.2957409266,-2.5740667573
Fe,0,-1.5125589864,-1.027335806,0.095757469
O,0,-1.0088900689,-2.2533607178,-1.4699604225
Fe,0,1.8740593964,-0.4837662704,-0.1326029743
O,0,1.2320665048,-2.0293829383,-1.4116475608
O,0,1.4493330413,-1.6505854026,1.5772753807
Fe,0,-0.4528509497,2.0484279119,-0.162876574
O,0,-0.1102586254,0.208116937,-0.060742025
C,0,0.1708240538,-2.4893593657,-1.8564240469
H,0,0.2503044264,-3.2040268693,-2.6962709062
C,0,0.4414504621,-2.2802263984,1.9301940258
H,0,0.5424699789,-2.9274717165,2.8199715569
N,0,3.6013609352,-1.0438719336,-0.0673128188
O,0,4.6345293371,-1.2341881416,0.4137586326
O,0,-3.2688086313,-2.3912232489,-0.0397074127
H,0,-2.9271004299,-2.991346563,-0.7178676929
H,0,-3.8780852821,-1.8128389196,-0.5194777664
O,0,-0.9870472345,4.2040958774,0.0398885332
H,0,-0.3017431341,4.4625439441,0.6722781579
H,0,-1.7862949393,4.1166962782,0.5778792886
```

$Fe_3O \cdot 2H_2O/1CO$, 2*S* +1 = 15

```
O,0,-2.0423582597,2.1509904581,1.3187271552
O,0,-2.7088561008,0.0145523282,1.5622908339
C,0,-2.7156946996,1.2318041633,1.8448244884
H,0,-3.3986663519,1.5352773706,2.6584581909
O,0,2.2524142155,0.8753123737,-1.5613326585
O,0,0.7625995714,2.5618653185,-1.6511612204
C,0,1.7908600313,1.9480190968,-2.0159112365
H,0,2.3609209481,2.4084970341,-2.8419779289
O,0,-0.7983074808,-2.1831248515,1.5823436772
O,0,0.8647437453,2.6891382446,1.2250449312
O,0,2.3118817005,0.9666822117,1.3162897513
C,0,1.8807295071,2.0916907002,1.6526857175
H,0,2.4679636298,2.6232037283,2.4234097259
O,0,-2.8573434876,-0.1287156796,-1.3150228498
O,0,-2.088018282,1.9727791376,-1.5612951564
C,0,-2.8437483574,1.014980721,-1.831623702
H,0,-3.5918347383,1.198433798,-2.6236701133
Fe,0,-1.6500269724,-1.0095903012,0.1593901086
O,0,-0.8833623992,-2.3337398136,-1.2922220893
Fe,0,1.5668909651,-0.3547888172,-0.0713131024
O,0,1.3057659044,-1.8391809522,-1.4684803994
O,0,1.407869504,-1.7655472354,1.4085742102
Fe,0,-0.5877925701,2.128488399,-0.1956511885
O,0,-0.2280378816,0.2557925626,-0.0355839033
C,0,0.268523957,-2.4709245663,-1.7680029797
H,0,0.3824346041,-3.2438072974,-2.5496474911
C,0,0.403576307,-2.3325299827,1.8989291951
H,0,0.6031578548,-3.0521212928,2.7124025065
C,0,3.8636163403,-1.136710664,-0.1097266921
O,0,4.9269973292,-1.4978532342,-0.1265422456
O,0,-3.2773652479,-2.4688756922,-0.0742300476
H,0,-2.8111402973,-3.0974733634,-0.6448340791
H,0,-3.8249270803,-1.953475868,-0.6849746471
O,0,-0.9870000919,4.2719875084,0.072185316
H,0,-0.2114167027,4.496960354,0.6062983285
H,0,-1.7041454036,4.1846672427,0.7180317146
```

$Fe_3O \cdot 3H_2O$, 2*S* +1 = 15

```
O,0,2.4173559726,1.3147084821,-1.3999751513
O,0,2.418724819,-0.9231116047,-1.6447870529
C,0,2.7802368207,0.2381338768,-1.9325661235
H,0,3.5088277692,0.3264962412,-2.758506475
O,0,-1.9980081923,1.3591017616,1.577508264
O,0,-0.0828125949,2.5430428751,1.6146573695
C,0,-1.2319728121,2.2477128722,2.0153989013
H,0,-1.6170582868,2.8448425497,2.8606131784
O,0,-0.0412126055,-2.4713515223,-1.6029715273
O,0,-0.1915177378,2.6871169659,-1.26741145
O,0,-2.0683705497,1.4456531886,-1.3446421815
C,0,-1.3338441103,2.394017025,-1.6956815395
H,0,-1.7417506123,3.0555618882,-2.4811946206
O,0,2.5848853169,-1.0801370992,1.2331411042
O,0,2.4655344756,1.1544694843,1.4788353148
C,0,2.917565882,0.019560219,1.7392464217
H,0,3.7073085123,-0.0214561875,2.510949964
Fe,0,1.1351030316,-1.5728883629,-0.2040101793
O,0,0.0643456185,-2.6222967321,1.2795326301
Fe,0,-1.7609381351,0.0099282199,0.0724320591
O,0,-1.8916438476,-1.528870275,1.4930403665
O,0,-2.0385438712,-1.4536945291,-1.3972278823
Fe,0,1.0426180881,1.7267968301,0.1360705492
O,0,0.1444943853,0.0428521765,0.0023965498
C,0,-1.0667453237,-2.4249993714,1.7805665729
H,0,-1.3747650372,-3.1307519771,2.5742255253
C,0,-1.2415727006,-2.2836902613,-1.8967928317
H,0,-1.6504918235,-2.929450046,-2.6946324326
O,0,2.2936196422,-3.4369134554,-0.0008743488
H,0,1.6906790453,-3.9148524171,0.5865838763
H,0,2.9770710097,-3.089201537,0.59133401
O,0,2.0622420324,3.6565769168,-0.1493571194
H,0,1.3805647165,4.1018883871,-0.6729566966
H,0,2.7088856942,3.3564118265,-0.805475028
```

```
O,0,-3.9373926739,-0.3032442618,0.1481723866
H,0,-4.0223011066,-0.9186301061,-0.5942662887
H,0,-4.005351781,-0.8546992605,0.939652955
```

Fe_3O-Cl, 2*S* +1 = 16

```
O,0,1.9795048187,1.5974802824,1.311156457
O,0,-0.0532704006,2.5432027387,1.5548696131
C,0,1.1661054491,2.3684677452,1.8449956165
H,0,1.5384896725,2.9867292409,2.6788069173
O,0,-0.0521141838,-2.5448876605,-1.5522708896
O,0,1.9794748398,-1.5951883134,-1.3138805136
C,0,1.1664634902,-2.3685270291,-1.8448450303
H,0,1.5384132992,-2.987642947,-2.678214152
O,0,-2.3580661602,0.9403419223,1.3442553441
O,0,1.9971500197,-1.297698708,1.5849995141
O,0,0.1917559512,-2.6049882359,1.253748872
C,0,1.2498563647,-2.2555107081,1.8468237522
H,0,1.5380527671,-2.8868162406,2.7042228986
O,0,0.1901880184,2.6072213664,-1.2507472845
O,0,1.9945557807,1.2998762952,-1.5872191086
C,0,1.2471229499,2.2583381756,-1.8462610437
H,0,1.5341020572,2.8909768195,-2.7030850379
Fe,0,-0.8987999601,1.5740541769,0.053490871
O,0,-2.2732913696,1.3079133316,-1.3982647262
Fe,0,-0.8979622724,-1.5744425697,-0.0521506844
O,0,-2.3574483046,-0.9416792482,-1.3434966552
O,0,-2.2734765331,-1.3092282836,1.3987305785
Fe,0,2.1959665337,0.0010425352,-0.0012366281
O,0,0.080170859,0.0000975765,-0.0000825605
C,0,-2.7068603553,0.1854351623,-1.7587770881
H,0,-3.4868659468,0.1992777271,-2.537407973
C,0,-2.70724635,-0.1868892898,1.7594271537
H,0,-3.4872295074,-0.2009963324,2.5380720232
Cl,0,4.3920424728,0.0018064704,-0.0024582356
```

Fe_3O-Cl/1NO, 2*S* +1 = 15

```
O,0,2.5150131532,0.9863937056,1.1658739505
O,0,1.0162336312,2.6285598256,1.5373780589
C,0,2.0962865896,2.0055292003,1.7406690328
H,0,2.7400666241,2.4299988859,2.5294080328
O,0,-1.139387801,-2.0827208547,-1.4819558555
O,0,1.1099726993,-1.9504263918,-1.3895416154
C,0,0.038193607,-2.3585540499,-1.8585838808
H,0,0.0999103848,-3.052964104,-2.7140054048
O,0,-1.727172245,2.0705867449,1.5194036725
O,0,1.4453455509,-1.686965863,1.5171746984
O,0,-0.7375598748,-2.2231387701,1.3545400899
C,0,0.4149550839,-2.2847689844,1.8669205843
H,0,0.5067574131,-2.9605003617,2.7345683004
O,0,1.0763034235,2.6274202614,-1.2801984364
O,0,2.198544336,0.7238249143,-1.7229265153
C,0,1.8701345047,1.9034627107,-1.9404719106
H,0,2.3259876272,2.3865658353,-2.8215095994
Fe,0,-0.2342602089,2.0469815943,0.1086884875
O,0,-1.6973062846,2.3624642638,-1.2434917998
Fe,0,-1.4848535903,-0.8848403682,0.0842967138
O,0,-2.6327759176,0.3133661956,-1.1789472627
O,0,-2.5363833251,-0.0298216878,1.6076813705
Fe,0,1.9860928748,-0.5508869033,-0.1194326092
O,0,0.034363614,0.2291814243,0.0174262638
C,0,-2.5357566921,1.4928886886,-1.5837672277
H,0,-3.2776222878,1.8131909796,-2.3349005324
C,0,-2.4633417352,1.1710494644,1.9748371016
H,0,-3.1318067998,1.4585194018,2.8037786451
N,0,-3.3934273663,-2.1131632535,0.0421034872
O,0,-4.2649523611,-1.8948675963,-0.6448583862
Cl,0,4.0107452123,-1.4047310377,-0.2748655137
```

Fe_3O-Cl/2NO, 2*S* +1 = 16

```
O,0,2.0387713607,-1.5916901915,-1.2911997823
O,0,0.0652000941,-2.661400639,-1.4872505181
C,0,1.2674399736,-2.4182147406,-1.7991344235
H,0,1.6648292666,-3.0273145676,-2.6291483515
O,0,-0.069105126,2.4870699257,1.5947015924
O,0,1.9948997304,1.6391672228,1.2840545832
C,0,1.1614458559,2.367901139,1.8484155345
H,0,1.5348750884,2.9969036213,2.6746875018
O,0,-2.2906426659,-1.1199465568,-1.3431326308
O,0,1.9325986595,1.2971646729,-1.6190511986
O,0,0.1231887441,2.587400099,-1.2447765588
C,0,1.1670663981,2.2456252167,-1.8643244515
H,0,1.4273070465,2.8743139754,-2.7333955053
O,0,0.320110399,-2.6086802349,1.3672200375
O,0,2.0781268818,-1.2220641169,1.6197194056
C,0,1.3683293285,-2.1916314445,1.932984585
H,0,1.6869027725,-2.7704368579,2.8170668656
Fe,0,-0.8312190525,-1.692442931,0.0215287814
O,0,-2.241510883,-1.3687710103,1.4627172124
Fe,0,-0.9402609127,1.5103178044,0.0754396568
O,0,-2.3832137491,0.8784709536,1.3977343547
O,0,-2.2791417595,1.1312076441,-1.4091193556
Fe,0,2.1858660972,0.0394995459,-0.0094707078
O,0,0.070445556,-0.0391951361,0.0204541632
C,0,-2.6978287631,-0.2533885029,1.8206229183
H,0,-3.4702485435,-0.2839009134,2.6078150329
C,0,-2.6534632694,-0.0055217188,-1.7816096399
H,0,-3.3961350921,-0.0236126963,-2.5978056592
N,0,-2.339996838,3.6964401953,0.1444504151
O,0,-1.980001428,4.777160933,0.063871054
N,0,-2.0483755187,-3.6184149918,-0.0675974272
O,0,-2.8969965751,-3.7866700928,-0.7965562983
Cl,0,4.3902209237,0.0939873941,-0.0595081859
```

Fe_3O-Cl/2NO, 2*S* +1 = 12

```
O,0,2.0951416051,-1.7070866923,-1.1372901367
O,0,-0.0096413941,-2.256444001,-1.7334675075
C,0,1.2372989491,-2.1800128639,-1.8989479079
H,0,1.5940262861,-2.6174916118,-2.8486849378
O,0,-0.105317571,2.5199662546,1.5011391396
O,0,1.9255292643,1.5983376354,1.2444779441
C,0,1.1148661172,2.3708899061,1.7810405662
H,0,1.4979806099,3.0074883741,2.5964156867
O,0,-2.5326059093,-0.8837405499,-1.2903711951
O,0,2.0319716335,1.0544961871,-1.6175520707
O,0,0.3391651424,2.5041920761,-1.3251463631
C,0,1.3671854619,2.064627282,-1.9052593219
H,0,1.7115327702,2.652691662,-2.7729909576
O,0,0.3380356224,-2.7013045076,1.2291398782
O,0,1.9373492965,-1.2015850382,1.754192117
C,0,1.2619141795,-2.2355497581,1.9360762322
H,0,1.5156712917,-2.8221305778,2.8378001641
Fe,0,-0.963368918,-1.7209917274,-0.0548192053
O,0,-2.0361341715,-1.1880349381,1.5890504652
Fe,0,-0.8683190974,1.5124465809,-0.0806770032
O,0,-2.4009999208,1.0067376378,1.2143817685
O,0,-2.1958501934,1.3232370827,-1.5935014898
Fe,0,2.1772660389,-0.0838079855,0.0930468617
O,0,-0.0278684029,-0.1569639858,-0.017786232
C,0,-2.5296984693,-0.0637110129,1.8430926254
H,0,-3.1701456211,-0.0331770269,2.7428242714
C,0,-2.7357355116,0.2106446125,-1.8430873943
H,0,-3.4849791965,0.2256407721,-2.6550214853
N,0,-2.076010311,3.4874590804,-0.0238175115
O,0,-3.0106442743,3.6351038758,0.5964324825
N,0,-1.8968138078,-3.208406643,-0.1330775353
O,0,-2.5257926236,-4.1571968081,-0.1776827152
Cl,0,4.3844751253,-0.0169902911,0.1704737676
```

Fe_3O-Cl/1CO, 2*S* +1 = 16

```
O,0,-2.5247453869,0.5477741708,-1.3375475359
O,0,-1.3645798648,2.4746767345,-1.4731347842
C,0,-2.2775704111,1.6693341754,-1.8105778583
H,0,-2.9294586041,2.0179393731,-2.6291450509
O,0,1.332111772,-1.8482115536,1.5781420191
O,0,-0.8827341292,-2.157168635,1.3011740073
C,0,0.2072543439,-2.3458619637,1.8645992052
H,0,0.2063616559,-3.0380759407,2.7240844067
O,0,1.4133534213,2.4019027542,-1.3402154074
O,0,-0.9855018471,-1.8987725089,-1.6197278933
O,0,1.2355182433,-2.0679379075,-1.2812051613
C,0,0.1532863763,-2.3153126163,-1.884072507
H,0,0.2434682836,-2.9889793637,-2.7534234892
O,0,-1.5321200164,2.3379995965,1.3367987176
O,0,-2.4006566203,0.2719830743,1.5660642369
C,0,-2.2640515674,1.4666513563,1.8807706123
H,0,-2.8522990408,1.8192856553,2.7449252944
Fe,0,-0.094064993,2.0437031995,-0.0106871642
O,0,1.2374742464,2.5530636502,1.4240120125
Fe,0,1.6573212721,-0.6179963044,0.0290214979
O,0,2.5459033675,0.7242790686,1.3177197975
O,0,2.5267325817,0.4518888104,-1.4913629422
Fe,0,-1.8534719884,-0.9110747845,-0.0296585262
O,0,-0.0384430933,0.1969361555,-0.005983031
C,0,2.221854573,1.8514705878,1.7566882098
H,0,2.8789804328,2.2806359301,2.5313397946
C,0,2.2812739055,1.6378616824,-1.8180238935
H,0,2.9051853803,2.0570397999,-2.6252673593
C,0,3.8612228959,-1.6348745984,0.0711968281
O,0,4.8794730085,-2.1067504271,0.0865943968
Cl,0,-3.718621197,-2.0803691708,-0.0708554328
```

Fe_3O-Cl/2CO, 2*S* +1 = 16

```
O,0,-1.8437993094,1.7857438403,-1.295555754
O,0,0.2701148582,2.5326639147,-1.5013025327
C,0,-0.9493171157,2.4709314795,-1.8179357385
H,0,-1.2495541187,3.1130069647,-2.6639474143
O,0,-0.2450160195,-2.5552414931,1.4856697583
O,0,-2.1653855538,-1.396988918,1.2843213748
C,0,-1.4284602902,-2.2527071791,1.800885424
H,0,-1.8541698619,-2.8294019862,2.6401303208
O,0,2.5109728211,0.828019659,-1.2716169536
O,0,-2.1064024937,-1.0570762523,-1.6329614751
O,0,-0.4413741193,-2.5521585173,-1.3794691597
C,0,-1.4619876506,-2.0712801274,-1.9450408615
H,0,-1.8188179294,-2.63046833,-2.8270962459
O,0,0.0379755548,2.5976307543,1.3579737841
O,0,-1.8777242251,1.4429380381,1.6189767373
C,0,-1.0562238185,2.3222257738,1.9239012915
H,0,-1.3046187628,2.9479972933,2.7983984377
Fe,0,1.073549786,1.5099427294,0.0328201433
O,0,2.3306067791,0.9337118624,1.5539521096
Fe,0,0.762859483,-1.6958251636,-0.0259930552
O,0,2.2831907394,-1.3012393966,1.3020470162
O,0,2.1308774787,-1.3744299084,-1.5264649718
Fe,0,-2.1627771164,0.2086174499,-0.0074707184
O,0,-0.0361247593,-0.0004828795,0.0029015883
C,0,2.6769364825,-0.23738068,1.8321068455
H,0,3.4186104907,-0.3442304568,2.6422012885
C,0,2.7018425239,-0.2927389773,-1.7961809362
H,0,3.4650467987,-0.3324823177,-2.5922000013
C,0,1.8657846667,-3.8838548796,-0.0671368159
O,0,2.3699988134,-4.887020188,-0.0857629748
C,0,2.5692956408,3.4478420625,0.0754014901
O,0,3.2486888696,4.3417861555,0.089034616
Cl,0,-4.3609026421,0.4208376735,-0.0176186173
```

Fe_3O-Cl·1H_2O, 2*S* +1 = 16

```
O,0,-1.4557149086,-1.7819549345,1.6371640541
O,0,0.7149867848,-2.3493796272,1.4369774331
C,0,-0.4259635453,-2.3924826455,1.9725488642
H,0,-0.5103532182,-3.0579813699,2.8484541868
O,0,-1.0683660542,2.4035025602,-1.4024327784
O,0,-2.2306169739,0.4921271696,-1.6859876865
C,0,-1.8732314474,1.6385324845,-2.0016727771
H,0,-2.3149652723,2.050653788,-2.9262469091
O,0,2.5305359988,-0.243908332,1.5723713172
O,0,-2.522365341,0.8643864342,1.2238127985
O,0,-1.0115509513,2.5200774602,1.4630352777
C,0,-2.074065314,1.8969648987,1.7450288414
H,0,-2.6740400103,2.3422419537,2.5574098508
O,0,1.0476724198,-2.3317011134,-1.3567750763
O,0,-1.1952735462,-2.1725671874,-1.2333426655
C,0,-0.1336624789,-2.6281119112,-1.6904500956
H,0,-0.2201051016,-3.3865178589,-2.4867684576
Fe,0,1.3804715571,-1.0242718107,0.1038501282
O,0,2.6337539762,-0.0671183952,-1.1968424074
Fe,0,0.289272842,1.9781562839,0.0551801796
O,0,1.745315525,1.983703939,-1.4472158706
O,0,1.8333366477,1.8895460565,1.3905684845
Fe,0,-2.0204810411,-0.7059339784,-0.0252748827
O,0,-0.0641753907,0.1202357093,0.0490800561
C,0,2.5648249489,1.0702910118,-1.709797285
H,0,3.3142409026,1.2980176587,-2.4871150307
C,0,2.5261163399,0.971198136,1.883489241
H,0,3.2181539557,1.2634482053,2.6908232015
Cl,0,-4.0689074983,-1.5165659562,-0.1146449966
O,0,0.7368587705,4.1145555507,-0.1821639202
H,0,1.2593937971,4.097010856,-0.9965076727
H,0,-0.0927293729,4.546765964,-0.4185364028
```

Fe_3O-Cl·1H_2O/1NO, 2*S* +1 = 15

```
O,0,-2.2217467146,-0.8498021673,1.6363610181
O,0,-0.7043846108,-2.4825286812,1.3103556346
C,0,-1.6733436866,-1.9321812102,1.901892388
H,0,-2.0789570296,-2.4921065858,2.7622789412
O,0,0.2765143923,2.7158502184,-1.123763761
O,0,-1.6841616842,1.6872185785,-1.546308476
C,0,-0.7906164722,2.5221200682,-1.7662875131
H,0,-0.9466119786,3.1848729847,-2.6362736648
O,0,2.0182405967,-1.6635598178,1.4654638701
O,0,-1.7444598639,1.9760876136,1.3840132001
O,0,0.4128024328,2.5222448108,1.7349321187
C,0,-0.8239887363,2.5570755931,1.9817836453
H,0,-1.1145274766,3.1934056806,2.8358139555
O,0,-0.4209376965,-2.4656819648,-1.5414571391
O,0,-2.2234201277,-1.1421476525,-1.2823409693
C,0,-1.5801524281,-2.0502716274,-1.8287263751
H,0,-2.0674315304,-2.5697442945,-2.671775907
Fe,0,0.5825555332,-1.6760859854,0.0127684715
O,0,2.1214510398,-1.3244516278,-1.3391180369
Fe,0,1.2125631576,1.4863520199,0.2215148186
O,0,2.4557851455,0.9002300792,-1.3513160017
O,0,2.5136962622,0.5336985058,1.4849352866
Fe,0,-2.1199651719,0.4548251653,0.0421698434
O,0,-0.0344658964,0.093683252,0.0900745825
C,0,2.6489677742,-0.2712235361,-1.7550471328
H,0,3.3760567597,-0.3854983812,-2.5784666134
C,0,2.6379106962,-0.6477728985,1.8691531559
H,0,3.3837516676,-0.8270481268,2.6624293946
N,0,1.4703245563,-3.7882870524,-0.1430824736
O,0,2.296565137,-4.0625639364,-0.8664983148
Cl,0,-4.2977789597,0.8339490388,-0.0223893183
O,0,2.7013436168,3.1226787177,0.1082728479
H,0,3.1246258957,2.8932562356,-0.731193026
H,0,2.1834551607,3.9150864739,-0.0793601098
```

Fe_3O-Cl·1H_2O/1CO, 2*S* +1 = 16

```
O,0,2.0129064661,-1.5269452375,-1.3688224743
O,0,0.0433164509,-2.6029673387,-1.5418467747
C,0,1.2302751278,-2.3419415379,-1.8810167784
H,0,1.6143206253,-2.9216055166,-2.7389482185
O,0,-0.1524341694,2.4701489596,1.5543938675
O,0,1.909794753,1.6085080401,1.287304738
C,0,1.0678718519,2.3358748589,1.8402707275
H,0,1.4248254462,2.9476721679,2.6867864987
O,0,-2.4092769328,-1.1885890738,-1.3206933222
O,0,1.8634876919,1.3238305474,-1.6332524641
O,0,0.0213187315,2.5769868054,-1.3068884938
C,0,1.0851067461,2.2531784429,-1.902639669
H,0,1.349354914,2.8812331501,-2.7709844336
O,0,0.274798065,-2.6538459642,1.3324116073
O,0,2.0172230351,-1.246663371,1.5691226881
C,0,1.3247741771,-2.226050053,1.886912732
H,0,1.6649367148,-2.8038896164,2.7643795504
Fe,0,-0.9420020325,-1.7026263872,0.0007597148
O,0,-2.2176093763,-1.3214115579,1.5507879604
Fe,0,-1.0414539598,1.512042041,0.0220210059
O,0,-2.4864183212,0.9026307455,1.3478754524
O,0,-2.3640615922,1.0557260978,-1.4809406831
Fe,0,2.1082107429,0.0453496149,-0.0361359774
O,0,-0.0229955595,-0.0584752755,-0.0072333489
C,0,-2.7181701751,-0.2168635544,1.860184524
H,0,-3.4529244175,-0.2308213036,2.6832889692
C,0,-2.760633106,-0.0882837686,-1.8027141325
H,0,-3.5117327665,-0.1313384691,-2.6099802352
C,0,-2.4234717622,3.5497838133,0.0391506474
O,0,-3.0475592686,4.4835107869,0.0466769431
Cl,0,4.3170233024,0.1349923603,-0.0666194462
O,0,-1.8818202396,-3.7065796791,0.0258078866
H,0,-1.4843455593,-4.1195492374,-0.7526568364
H,0,-1.4463058536,-4.1199130801,0.7833170351
```

Fe_3O-Cl·2H_2O, 2*S* +1 = 16

```
O,0,-1.4801271446,-1.7381522702,1.6434432943
O,0,0.6774414998,-2.3770648088,1.5483657227
C,0,-0.4811706425,-2.356798311,2.0458897757
H,0,-0.6143925675,-2.9715345328,2.9528352345
O,0,-0.9795739467,2.3664897598,-1.4936681616
O,0,-2.1309115145,0.4531514349,-1.7738074088
C,0,-1.7849976805,1.6011021022,-2.0926657279
H,0,-2.2340799748,2.013212484,-3.0135258534
O,0,2.5319661443,-0.1697119274,1.6734311046
O,0,-2.4376164539,0.9196283989,1.1227997889
O,0,-0.8726613976,2.5154730284,1.381266127
C,0,-1.9600723251,1.937063749,1.6503059041
H,0,-2.5602407684,2.40434904,2.4509734612
O,0,1.034990642,-2.431634237,-1.3136742029
O,0,-1.202453957,-2.2020294177,-1.2530493865
C,0,-0.1438059281,-2.6806960542,-1.6879646392
H,0,-0.2353491486,-3.4218860337,-2.5014349481
Fe,0,1.4997065267,-1.1500203271,0.2151306811
O,0,2.7638068583,-0.172019739,-1.1566205427
Fe,0,0.4096412803,1.8787225352,-0.0788606757
O,0,1.8032474827,1.819222062,-1.5718307234
O,0,1.9003089413,1.9606249474,1.3131887713
Fe,0,-1.9495914745,-0.6912350814,-0.0657703081
O,0,0.0503927138,0.0374245579,0.0248236897
C,0,2.645794864,0.9158368459,-1.7670833998
H,0,3.3744914943,1.1026031311,-2.5754966507
C,0,2.5406194817,1.0614713978,1.9030780077
H,0,3.1962432718,1.3943649092,2.7260548479
Cl,0,-4.0291012923,-1.4451220226,-0.1563530593
O,0,0.6935102502,4.0772391708,-0.2151677391
H,0,0.1062214308,4.2871666753,-0.9541279259
H,0,0.2302715384,4.3894579952,0.573302823
O,0,3.2675787091,-2.4878198244,0.1689098151
H,0,3.9376186896,-1.9189493794,-0.2326784897
H,0,2.9971053574,-3.0846720984,-0.5431638159
```

Fe_3O-OH, 2*S* +1 = 16

```
O,0,-1.6740527482,1.6625657509,-1.2840101022
O,0,0.3619355871,2.6280883869,-1.419928099
C,0,-0.8476679086,2.4559883765,-1.7571509811
H,0,-1.1894569509,3.1061139191,-2.5809229134
O,0,0.3295803459,-2.5554463939,1.4980056509
O,0,-1.6848112554,-1.5431739097,1.3999700824
C,0,-0.8668504477,-2.3535898579,1.8608841913
H,0,-1.2087333324,-2.9892570756,2.6952896023
O,0,2.6508056527,0.9969244839,-1.3351033612
O,0,-1.7548068325,-1.2486071327,-1.5478215789
O,0,0.0704283316,-2.5505794226,-1.3126863923
C,0,-1.0131968375,-2.1941470907,-1.8574754819
H,0,-1.3253794418,-2.8134199888,-2.716201116
O,0,0.2083863763,2.5911085052,1.3996967554
O,0,-1.6647725669,1.3654013991,1.6657843048
C,0,-0.8774261086,2.2780985512,1.96400326
H,0,-1.1489526758,2.9034515218,2.8315327219
Fe,0,1.2273782668,1.5888490063,0.0167101819
O,0,2.6356618377,1.2290355488,1.4204649505
Fe,0,1.174136842,-1.5650866811,0.0092687363
O,0,2.6710083434,-1.0199916773,1.2992894615
O,0,2.5335491084,-1.249695153,-1.4539590921
Fe,0,-1.8803041264,0.0610956446,0.0846782756
O,0,0.2292608648,0.0283242016,0.0201632047
C,0,3.0519560765,0.0878609541,1.7384099463
H,0,3.8482393213,0.06256463,2.5004881969
C,0,2.974485137,-0.1229565105,-1.7895188736
H,0,3.7378923563,-0.1237941391,-2.5850357673
O,0,-3.7024980897,0.0915419393,0.0564744908
H,0,-4.1568899453,0.0986053337,-0.7908472445
```

Fe_3O-OH/1CO, 2*S* +1 = 16

```
O,0,-2.5516690079,0.7148265245,-1.2499982197
O,0,-1.2720103865,2.5632000746,-1.4209362049
C,0,-2.2426503359,1.8153922649,-1.7332524367
H,0,-2.8859012133,2.2067152864,-2.5404857547
O,0,1.2648599662,-1.910795807,1.5617958077
O,0,-0.976728623,-2.0819833439,1.4004031313
C,0,0.1272458498,-2.3403831973,1.9047522424
H,0,0.130347017,-3.0363970714,2.7614881035
O,0,1.500494461,2.3588900586,-1.3692387329
O,0,-1.1429540531,-1.8204672124,-1.5672988263
O,0,1.0783687015,-2.0969855676,-1.2994999666
C,0,-0.0356345567,-2.2862918051,-1.8701723107
H,0,0.0018058855,-2.9570045536,-2.746675231
O,0,-1.3548344724,2.4675423322,1.3951856843
O,0,-2.3320020435,0.4593059748,1.6988520572
C,0,-2.1159295904,1.6483039703,1.9826203052
H,0,-2.65088859,2.0548899103,2.8581670314
Fe,0,0.0161945976,2.0753197251,0.0067907507
O,0,1.4259462249,2.4977666202,1.3990668138
Fe,0,1.6132095641,-0.6903683871,0.0084300504
O,0,2.6127463085,0.5869050129,1.2911242329
O,0,2.5114763933,0.3526194325,-1.519655752
Fe,0,-1.8901705084,-0.771811902,0.0944444001
O,0,-0.024184368,0.2306312446,0.0190023056
C,0,2.3685706212,1.7368804284,1.7219099037
H,0,3.065403163,2.1309629993,2.4807910409
C,0,2.320840175,1.5468111372,-1.8518524321
H,0,2.9531821093,1.9273311667,-2.6720643082
C,0,3.7626295996,-1.8126878672,0.0018762806
O,0,4.7586204986,-2.3304347101,0.0063882906
O,0,-3.497685165,-1.6374942293,0.060356886
H,0,-3.9904942119,-1.6501815994,-0.7652947424
```

Fe_3O-OH/2CO, $2S+1 = 16$

```
O,0,2.1155836667,-1.494283936,-1.3015153986
O,0,0.1414815512,-2.5647287014,-1.4835195163
C,0,1.3346358388,-2.313130583,-1.8107210201
H,0,1.7193696778,-2.9074931436,-2.6585711065
O,0,-0.1313743116,2.5149338223,1.5553537227
O,0,1.9384536347,1.6392709984,1.4100253677
C,0,1.0734232059,2.3800541661,1.9046212092
H,0,1.3878351398,3.0073285883,2.7569460793
O,0,-2.3293389881,-1.194902806,-1.3384969681
O,0,1.9589084487,1.3700076189,-1.562748051
O,0,0.0843455519,2.5937204484,-1.3096698074
C,0,1.1735598782,2.2781909224,-1.8692741385
H,0,1.446398908,2.9018445286,-2.7390560586
O,0,0.2649455669,-2.611204397,1.3895985701
O,0,2.025532284,-1.2355859539,1.6790888222
C,0,1.311341392,-2.2056314584,1.9712095097
H,0,1.6176667258,-2.8018880443,2.8486362763
Fe,0,-0.8538085215,-1.6745295381,0.0176742988
O,0,-2.2400596463,-1.2896768378,1.4917310517
Fe,0,-0.9952024441,1.5505299631,0.0128097103
O,0,-2.4663750899,0.9429573502,1.3197089364
O,0,-2.2917197787,1.0495833681,-1.5063940581
Fe,0,2.1516224032,0.082834499,0.0832857079
O,0,0.0210951213,-0.0207084969,0.024622367
C,0,-2.7370203601,-0.1819227931,1.7984169557
H,0,-3.5080121071,-0.1992364122,2.5881800803
C,0,-2.6792934637,-0.0970522005,-1.8282337421
H,0,-3.4205876355,-0.1454949202,-2.6447560513
C,0,-2.3742245339,3.5692505592,-0.0119445513
O,0,-2.9921025929,4.5070026588,-0.0210797816
C,0,-2.0546170528,-3.8044580387,0.0120290176
O,0,-2.59282036,-4.7901501328,0.0104866243
O,0,3.9806818236,0.1448089768,0.0563974443
H,0,4.4181896279,-0.0925236846,-0.7666156417
```

Fe_3O-OH/1NO, $2S+1 = 15$

O	2.77392200	0.26633600	1.22405600
O	1.75480600	2.27235700	1.38409400
C	2.62550700	1.40750900	1.68973200
H	3.33769100	1.72293500	2.47138000
O	-1.53508800	-1.92596200	-1.35256100
O	0.67916900	-2.36030400	-1.28522400
C	-0.46872000	-2.50560800	-1.72330100
H	-0.60661700	-3.23003100	-2.54547000
O	-1.03673900	2.43370500	1.35488200
O	1.04591600	-2.04760600	1.66518500
O	-1.20062400	-1.97573800	1.49888400
C	-0.10782300	-2.32578000	2.02711200
H	-0.20601500	-2.96254300	2.92346200
O	1.81001700	2.06590300	-1.43155200
O	2.45473900	-0.07700400	-1.70544300
C	2.41898300	1.12543900	-2.01403800
H	2.98873200	1.42607100	-2.91085200
Fe	0.40275900	1.93125500	-0.02757300
O	-0.93814500	2.52450300	-1.41583900
Fe	-1.56131300	-0.58165100	0.12259600
O	-2.36617600	0.78988800	-1.23428900
O	-2.35862300	0.62141700	1.56818500
Fe	1.87002500	-1.15131900	-0.00073000
O	0.18722200	0.10621500	0.00970100
C	-1.97231000	1.87732300	-1.70999100
H	-2.61155600	2.32854800	-2.48825900
C	-1.97790100	1.78510400	1.85634500
H	-2.54844200	2.28930800	2.65485000
N	-3.72581800	-1.26107400	0.12945900
O	-4.51112100	-0.86045300	-0.57971700
O	3.30555000	-2.27444900	-0.10571700
H	3.60368500	-2.55655200	-0.97523800

Fe_3O-OH/2NO, 2*S* +1 = 16

O	1.51887000	2.21585900	-1.33308900
O	2.70766800	0.30012400	-1.36371800
C	2.41537000	1.46959300	-1.75069400
H	3.05068800	1.85922800	-2.56506500
O	-2.57658700	-0.03702900	1.45311600
O	-1.79704200	2.06346600	1.19993300
C	-2.51865900	1.19715100	1.71868500
H	-3.20617300	1.53296100	2.51519200
O	1.30387500	-2.13692100	-1.23730500
O	-1.35645700	1.94110300	-1.74621500
O	-2.52944800	0.04821300	-1.39611000
C	-2.23374000	1.10579300	-2.01892300
H	-2.84119800	1.30372100	-2.91951900
O	2.51753100	0.59522800	1.48374100
O	1.05677700	2.30689200	1.61754300
C	2.03240200	1.64330100	1.99812300
H	2.55856400	2.00252100	2.90037100
Fe	1.72417700	-0.62089700	0.12295900
O	1.39077200	-2.02142800	1.57692400
Fe	-1.47232100	-0.89861700	0.02250300
O	-0.84375400	-2.27002700	1.42256000
O	-0.93974900	-2.26535900	-1.39312200
Fe	-0.15363100	2.28191500	-0.09975700
O	0.02688700	0.18333300	0.02597100
C	0.28276000	-2.51911600	1.90003400
H	0.31459100	-3.26836900	2.70971800
C	0.23188000	-2.57900000	-1.70771400
H	0.32876600	-3.34187200	-2.49975600
N	-3.58780700	-2.41385800	0.02520500
O	-4.67802600	-2.11338200	-0.13471700
N	3.71154200	-1.73280200	0.15316900
O	3.96230300	-2.58120600	-0.55275500
O	-0.30754300	4.10349300	-0.13529300
H	-0.53393000	4.55081600	0.68535500

Fe_3O-OH/2NO, 2*S* +1 = 14

O	-2.35269800	1.55191700	1.16606500
O	-2.30534500	-0.65948900	1.61901000
C	-2.60452900	0.54153800	1.84413000
H	-3.18786000	0.69818700	2.77016500
O	2.46322800	0.92093100	-1.31967900
O	0.90895700	2.53848300	-1.17419700
C	1.94793900	2.03411000	-1.62377100
H	2.50250100	2.61442100	-2.38175300
O	-0.10307200	-2.56828200	1.23470500
O	0.29911000	2.40133100	1.70959700
O	2.18431400	1.19809600	1.45898200
C	1.44843400	2.05349100	2.02290100
H	1.89423400	2.54724500	2.90377900
O	-2.62881600	-0.38770300	-1.37417400
O	-1.73765000	1.64710600	-1.76914200
C	-2.46959900	0.67066700	-2.02761600
H	-3.06660600	0.74739100	-2.95553400
Fe	-1.39864100	-1.36120800	-0.04664600
O	-0.38109200	-2.16735800	-1.66474800
Fe	1.60735600	-0.18338200	0.13202700
O	1.78290800	-1.72509300	-1.20712000
O	1.88531700	-1.56357600	1.56659900
Fe	-0.81521900	2.16783500	-0.01704700
O	-0.17640600	0.08010800	0.03524900
C	0.84897300	-2.23386700	-1.87165400
H	1.15101200	-2.82526000	-2.75529200
C	1.00568400	-2.44624100	1.77838000
H	1.27375900	-3.19611800	2.54464100
N	4.23098800	-0.68020300	0.12603900
O	4.77387000	-1.41066400	-0.53963500
N	-2.43760600	-2.80062300	-0.03186900
O	-2.91361500	-3.83557700	0.06681200
O	-1.34138000	3.91800700	-0.10070400
H	-1.27403300	4.35149300	-0.95703200

Fe_3O-OH/2NO, 2*S* +1 = 12

O	-2.32955200	1.52257300	1.19637100
O	-2.18750200	-0.68048200	1.66771500
C	-2.51755900	0.51563700	1.89487300
H	-3.06449800	0.65616300	2.84512400
O	2.43783300	0.91634000	-1.41147700
O	0.88670900	2.52881200	-1.19318000
C	1.90118800	2.02277000	-1.69657900
H	2.40774700	2.59913300	-2.49033100
O	-0.04102500	-2.58576400	1.21273900
O	0.30316800	2.38074400	1.70854200
O	2.21927500	1.24226700	1.41630200
C	1.46737000	2.06555000	2.00386800
H	1.90717000	2.56118300	2.88645900
O	-2.64255600	-0.39560700	-1.30096000
O	-1.75900400	1.62958900	-1.75286600
C	-2.49829500	0.65106700	-1.97752400
H	-3.11831800	0.71209200	-2.89156500
Fe	-1.32532600	-1.34478600	-0.01572700
O	-0.41896900	-2.13287400	-1.66270500
Fe	1.69830600	-0.18136400	0.11496000
O	1.76320600	-1.75693500	-1.22668200
O	1.92083700	-1.54584200	1.59481800
Fe	-0.80130800	2.13982800	-0.01565700
O	-0.15351400	0.05750500	0.01836500
C	0.81085200	-2.21764600	-1.88927500
H	1.07473800	-2.78831000	-2.79808300
C	1.04550300	-2.43170000	1.79539500
H	1.29044200	-3.15942400	2.59029200
N	3.95993600	-0.68163000	0.10503700
O	4.42128700	-1.49064900	-0.53803100
N	-2.41536900	-2.72645600	-0.00622900
O	-3.08053100	-3.65249000	-0.00276800
O	-1.35277200	3.88411200	-0.08234100
H	-1.32221100	4.31567000	-0.94178500

Fe_3O-OH·1H_2O, 2*S* +1 = 16

```
O,0,2.481780736,1.0782125953,-1.3701004509
O,0,2.3930029026,-1.1675623634,-1.5334042196
C,0,2.7810576289,-0.0149167846,-1.8738946193
H,0,3.4817871626,0.0102107893,-2.7272841466
O,0,-2.1613675066,1.2613562897,1.4480588103
O,0,-0.3209125841,2.5574521918,1.3249440471
C,0,-1.426166909,2.2315021928,1.7871533003
H,0,-1.8330328914,2.8583796062,2.5997166352
O,0,-0.108395118,-2.5427985996,-1.3447560263
O,0,-0.0757793958,2.3961778792,-1.6286881378
O,0,-2.0659678209,1.3704546341,-1.3666872984
C,0,-1.2568868212,2.1608537843,-1.9290232572
H,0,-1.6661304895,2.7056030515,-2.7975329746
O,0,2.5263512876,-1.0514900435,1.3475453125
O,0,2.2233590615,1.1664955306,1.6161363915
C,0,2.690043272,0.0621925478,1.9262485573
H,0,3.3394698745,0.030013511,2.8197677366
Fe,0,1.1141556512,-1.5975044754,-0.0039608475
O,0,0.0776396428,-2.4889015022,1.5131594538
Fe,0,-1.6494042232,-0.0077236768,0.0082466894
O,0,-1.9416709375,-1.5031226353,1.3679372788
O,0,-2.0523357358,-1.4064866655,-1.4041474453
Fe,0,1.1661210824,1.9298507947,-0.0331656064
O,0,0.1931659511,0.0501489329,0.0029393597
C,0,-1.1177275909,-2.314319743,1.8470716576
H,0,-1.4889557579,-2.9542431294,2.6655829761
C,0,-1.2660542867,-2.3142688045,-1.7629174412
H,0,-1.6418698141,-2.9939940842,-2.5462336862
O,0,2.3344725092,-3.4262069102,-0.0102844548
H,0,2.8957716073,-3.2911725846,-0.7857836325
H,0,2.9136732303,-3.2895469814,0.7514201586
O,0,2.011171246,3.5490105621,0.0329747968
H,0,1.9623470366,4.0489720901,0.852981083
```

Fe_3O-OH·1H_2O/1CO, $2S+1 = 16$

```
O,0,2.5359834635,0.3099263376,1.3991175386
O,0,1.3822514701,2.237070249,1.5522733601
C,0,2.2778776795,1.4174068496,1.8954188268
H,0,2.9106024496,1.7381732208,2.7421165176
O,0,-1.3870965756,-2.0661225043,-1.4888514292
O,0,0.8382689878,-2.3542832437,-1.2919411799
C,0,-0.2723026645,-2.5654898394,-1.8049553171
H,0,-0.3041495974,-3.2821447676,-2.6449754287
O,0,-1.4430273035,2.2937095683,1.3512295717
O,0,0.9205089123,-2.0620579446,1.686986898
O,0,-1.3089034411,-2.1721072196,1.3807130077
C,0,-0.2249596625,-2.4412579216,1.9718624751
H,0,-0.3249740222,-3.1008976851,2.851909309
O,0,1.5842749585,2.175244484,-1.3249512518
O,0,2.3669214898,0.0785285308,-1.5845902897
C,0,2.2627508207,1.272634157,-1.8954291667
H,0,2.8332241754,1.6081301553,-2.7805354243
Fe,0,0.0625135488,1.9657816392,0.0116328514
O,0,-1.2487087104,2.2810753478,-1.5239293463
Fe,0,-1.6675059607,-0.7547654644,0.0093234605
O,0,-2.5927092342,0.4836479511,-1.3505908147
O,0,-2.5789271449,0.3546956572,1.4845126069
Fe,0,1.7808009891,-1.0714129978,0.0763021936
O,0,0.0017259647,0.0868760536,0.0194493184
C,0,-2.2286692391,1.573412932,-1.8491408839
H,0,-2.848477264,1.9579665929,-2.677590446
C,0,-2.3220442538,1.5344811505,1.817507391
H,0,-2.9482806889,1.9575385029,2.6220048813
C,0,-3.8990793623,-1.7799514367,0.013424699
O,0,-4.9171617702,-2.2543568678,0.0235749495
O,0,0.2848850622,4.1721659048,-0.0144693323
H,0,0.8162945594,4.3154071889,0.7803389496
H,0,0.9001107358,4.279845917,-0.7526685235
O,0,3.3053111924,-2.0858988989,0.0443662731
H,0,3.4838539059,-2.5833531589,-0.7591852553
```

Fe_3O-OH·1H_2O/1NO, $2S+1 = 15$

```
O          -0.52936200   -2.40375000    1.60082700
O           1.64838700   -1.84023700    1.46770900
C           0.65096700   -2.42359600    1.97964300
H           0.87648100   -3.02657500    2.87725300
O          -2.19946000    1.52197400   -1.36833400
O          -2.25107700   -0.69495600   -1.77493600
C          -2.51456600    0.49055800   -2.02588700
H          -3.10370200    0.68024100   -2.94151300
O           2.25145800    0.94085400    1.58029500
O          -2.74379900   -0.60825700    1.18765400
O          -2.18108200    1.55060400    1.50960400
C          -2.83167100    0.49548300    1.74852300
H          -3.57555800    0.58328000    2.56054900
O           1.98252600   -1.68312600   -1.37727000
O          -0.08211300   -2.58325200   -1.33662900
C           1.07974200   -2.48306800   -1.75709700
H           1.37929600   -3.17271100   -2.56543200
Fe          1.67438300   -0.37248000    0.11939000
O           2.24323800    1.09598600   -1.23837200
Fe         -0.78323800    1.73363600    0.09831100
O           0.48808100    2.49683400   -1.38946500
O           0.57671400    2.44354800    1.45740000
Fe         -1.48991100   -1.67453300   -0.11762000
O          -0.16433500   -0.03004200    0.03639800
C           1.62360500    2.07285800   -1.71009700
H           2.14174600    2.63422400   -2.50821600
C           1.64204300    1.98488600    1.92079100
H           2.11006100    2.56784500    2.73286100
N           3.95533200   -0.64501700    0.16150300
O           4.66043100   -0.08551600   -0.52566000
O          -1.46671900    3.83228900   -0.14085500
H          -0.86763300    4.10030900   -0.85242500
H          -2.31946200    3.70488700   -0.57735800
O          -2.66701300   -3.07559400   -0.18774100
H          -3.18831600   -3.25017700    0.60140600
```

Fe_3O-OH·$2H_2O$, 2*S* +1 = 16

```
O,0,2.3692106215,1.0524337719,-1.3912159094
O,0,2.2746909789,-1.1907480135,-1.5392600744
C,0,2.6630698669,-0.0440956031,-1.8920661229
H,0,3.355940632,-0.0238994523,-2.7521785203
O,0,-2.1282221544,1.2749791898,1.6397563732
O,0,-0.3211391281,2.5918321999,1.3657890174
C,0,-1.3752597348,2.2462130233,1.9234098814
H,0,-1.7121697305,2.8620377601,2.7764156569
O,0,-0.1633593498,-2.5937077481,-1.2907574336
O,0,-0.1878702952,2.3406982987,-1.6245505582
O,0,-2.2025630309,1.4125324362,-1.2306066094
C,0,-1.3877089284,2.1442348394,-1.8617131479
H,0,-1.8124419802,2.6727529576,-2.7343915605
O,0,2.4900450391,-1.042208394,1.3359192334
O,0,2.1866270437,1.1747361139,1.5963689216
C,0,2.6724503958,0.0761841478,1.8976044796
H,0,3.3576323084,0.0560782695,2.7645518087
Fe,0,1.0176058523,-1.579991936,0.0349134877
O,0,0.0710439929,-2.5128822078,1.5869927949
Fe,0,-1.8198051687,-0.0337729619,0.1646262676
O,0,-1.9777862691,-1.5874581469,1.4716580646
O,0,-2.0980433433,-1.4477558439,-1.3720053424
Fe,0,1.0759950231,1.8948269897,-0.0360064778
O,0,0.0562583365,0.0306154211,0.0636187367
C,0,-1.1191907748,-2.3665563798,1.9452009911
H,0,-1.4551949838,-3.0047183355,2.7806955975
C,0,-1.3088832957,-2.3535078429,-1.7294493442
H,0,-1.669110646,-3.023819363,-2.5298647633
O,0,2.2802740184,-3.4090387803,0.0113599578
H,0,2.787028821,-3.2634702322,-0.7991569567
H,0,2.8941764278,-3.2109078374,0.7315249136
O,0,1.9243323936,3.5196312656,-0.0365816072
H,0,1.8336532072,4.05499336,0.7573614975
O,0,-4.0306053775,-0.1654031636,-0.0272634801
H,0,-4.2042341313,0.6521211294,-0.5139763569
H,0,-4.0864299866,-0.8605457517,-0.6981831957
```

References

1. Leclerc, H.; Vimont, A.; Lavalley, J.-C.; Daturi, M.; Wiersum, A. D.; Llwellyn, P. L.; Horcajada, P.; Ferey, G.; Serre, C., Infrared study of the influence of reducible iron(iii) metal sites on the adsorption of CO, CO2, propane, propene and propyne in the mesoporous metal-organic framework MIL-100. *Phys. Chem. Phys. Chem.* **2011,** *13* (24), 11748-11756.
